# Determining therapeutic susceptibility in multiple myeloma by single-cell mass accumulation

**DOI:** 10.1038/s41467-017-01593-2

**Published:** 2017-11-20

**Authors:** Arif E. Cetin, Mark M. Stevens, Nicholas L. Calistri, Mariateresa Fulciniti, Selim Olcum, Robert J. Kimmerling, Nikhil C. Munshi, Scott R. Manalis

**Affiliations:** 1 Koch Institute for Integrative Cancer Research, Massachusetts Institute of Technology, 500 Main St., Cambridge, MA 02139 USA; 2 Department of Medical Oncology, Dana-Farber Cancer Institute, Harvard Medical School, 450 Brookline Ave., Boston, MA 02215 USA; 3 Jerome Lipper Multiple Myeloma Center, Department of Medical Oncology, Dana-Farber Cancer Institute, 450 Brookline Ave., Boston, MA 02215 USA; 4 Veterans Affairs Boston Healthcare System, Harvard Medical School, 150 S Huntington Ave., Boston, MA 02130 USA; 5 Department of Biological Engineering, Massachusetts Institute of Technology, 77 Massachusetts Ave., Cambridge, MA 02139 USA; 6 Department of Mechanical Engineering, Massachusetts Institute of Technology, 77 Massachusetts Ave., Cambridge, MA 02139 USA

## Abstract

Multiple myeloma (MM) has benefited from significant advancements in treatment that have improved outcomes and reduced morbidity. However, the disease remains incurable and is characterized by high rates of drug resistance and relapse. Consequently, methods to select the most efficacious therapy are of great interest. Here we utilize a functional assay to assess the ex vivo drug sensitivity of single multiple myeloma cells based on measuring their mass accumulation rate (MAR). We show that MAR accurately and rapidly defines therapeutic susceptibility across human multiple myeloma cell lines to a gamut of standard-of-care therapies. Finally, we demonstrate that our MAR assay, without the need for extended culture ex vivo, correctly defines the response of nine patients to standard-of-care drugs according to their clinical diagnoses. This data highlights the MAR assay in both research and clinical applications as a promising tool for predicting therapeutic response using clinical samples.

## Abstract (web summary)

Multiple myeloma is characterized by high rates of drug resistance and relapse. Here the authors utilize a functional assay to assess the ex vivo drug sensitivity of single multiple myeloma cells based on measuring the mass accumulation rate of individual cells.

## Introduction

Multiple myeloma (MM) is characterized by the accumulation of clonal plasma cells in the bone marrow^1, 2^. Therapeutic advances have greatly reduced the morbidity and mortality in this disease through the incorporation of novel-targeted agents such as proteasome inhibitors, (e.g. bortezomib and carfilzomib)^3^, immunomodulatory drugs (lenalidomide, pomalidomide)^4^, novel antibodies (daratumumab and elotuzumab)^5, 6^, and HDAC inhibitors in a treatment regimen that includes traditional chemotherapeutic agents and high-dose therapy with stem cell transplants^7^. Despite these advances, MM remains incurable in the vast majority of patients although there is a high degree of variability in patient survival. This variability is in part due to the heterogeneity of the disease at the molecular, clonal, and cellular level, which affects MM cells’ susceptibility and resistance to therapies^8–12^.

Today, most approaches—especially in solid tumors—define therapeutic susceptibility based on the presence or absence of genetic or epigenetic markers^13^. However, these approaches have had limited success, primarily due to two factors: a lack of validated biomarkers, and an inability of these bulk assays to identify and probe the response of small resistant subpopulations. Existing biomarkers are validated based on response across large patient populations, which weakens their reliability as predictors of individual patient response, particularly following relapse post treatment with biomarker-specified therapy^14, 15^. Single-cell sequencing can resolve cellular heterogeneity, but this approach still requires previously defined genetic markers and suffers from persistent issues concerning throughput^16^.

In contrast to these genetic and epigenetic approaches, functional assays aim to offer a direct measurement of therapeutic response providing a phenotype-based evaluation of drug susceptibility using patient cells. For therapeutic susceptibility assays, a functional biomarker is a measurable, integrative parameter of all genetic, epigenetic, and environmental cues that affect cells’ therapeutic susceptibility^17^. Functional assays are already key to patient care decisions, where measurement of patient disease burden by imaging or direct quantification from the peripheral blood is used as a retrospective, treatment guiding indicator of therapeutic response. Ideally, however, functional assessment would occur prior to therapy selection and administration of drug to the patient, thereby preventing the patient morbidity and mortality associated with selection of inefficacious drugs.

The difficulties facing functional testing of drug susceptibility in cancer are distinct from their genomic biomarker-based counterparts. Despite their long-term, widespread use for in vitro studies, there has yet to be a prospective, in vitro functional assay routinely applied in the clinic. Historically, functional assays are limited by a variety of factors including requirements for large tissue samples, artifact-inducing long-term cell culture, and bulk measurement approaches. These requirements are complicated further by a lack of ex vivo primary cell proliferation in most diseases, including MM. Despite these difficulties, the appeal of functional indicators of drug susceptibility that are treatment agnostic has encouraged continued development. Recent progress in single-cell functional assays have mitigated some of these shortcomings and show promise for identification and targeting of subpopulations of response on small samples^18–20^.

We recently introduced an approach to functionally assess single-cell therapeutic susceptibility by determining mass accumulation rate (MAR) and mass of single cancer cells^18, 21–24^. Using a microfluidic device known as the suspended microchannel resonator (SMR), we measured the mass of individual cells repeatedly over 15–20 min intervals to define single-cell MARs. In acute lymphocytic leukemia and glioblastoma models, we previously showed that MARs of single, sensitive cells are reduced when measured following exposure to targeted small-molecule therapies, whereas cells with a resistance mutation to that therapy will maintain MARs matching that of control conditions^25^.

**Fig. 1:**
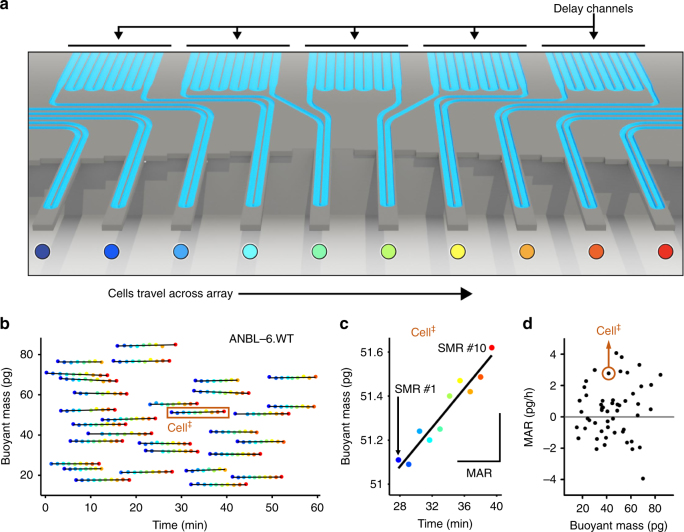
MAR measurements characterize heterogeneity in cell growth across human MM cell lines. Serial suspended microchannel resonator (sSMR) workflow schematics: **a** Cells pass through the sSMR device. Each cell is weighed multiple times over a 15-min interval in the sSMR device, which consists of multiple sensors that are fluidically connected in series and separated by delay channels (not all is shown). This design enables a stream of cells to flow through the device such that different sensors can concurrently weigh flowing cells in the stream, revealing single-cell MARs. **b** Real-time and high-throughput monitoring of mass change for ANBL-6.WT cells with sSMR device. **c** The change in the cell mass highlighted with a box in Fig. 1b, where blue (SMR #1) to red (SMR #10) dots correspond to the mass of the same cell sequentially measured with 10 different sensors. MAR is calculated from the slope of the linear fit applied to the mass vs. time data. **d** MAR calculated from each individual cell trace is plotted with respect to the buoyant mass of each cell. The cell highlighted in Fig. 1b is denoted with a circle. Number of cells in MAR measurement; *n* = 53

Here we demonstrate the capability of this assay to functionally assess the therapeutic sensitivity of single MM cells to standard-of-care (SOC) and experimental therapies. Utilizing a high-throughput MAR measurement platform known as the serial SMR (sSMR) (Fig. 1a)^25^, we validate the MAR response of human MM cell lines and primary patient samples when treated with SOC therapies including dexamethasone, lenalidomide, and bortezomib. We show MAR is reduced in response to SOC therapies administered alone and that combinations of therapies resulted in larger magnitude reductions in MAR. Additionally, we observe MAR response when using a peptide-based therapeutic approach for targeting E2F/DP1 interaction in combination with BET inhibitor JQ1. This indicates the compatibility of the MAR assay with peptide-based therapeutics and also suggests that functional MAR measurements could potentially assess and select for patient sensitivity to a range of experimental MM therapies.

## Results

### MAR measures single-cell growth heterogeneity in multiple myeloma

The SMR is a microfluidic mass sensor, capable of measuring buoyant mass (hereafter referred to as “mass”) of single, live cells as they flow through a suspended microchannel with a precision of ~50 fg, roughly 3 orders of magnitude less than the total mass of a cell^18, 23, 24^. The serial SMR consists of an array of these sensors that are fluidically connected in series and separated by delay channels (Fig. 1a). Cells flowing through this array take ~1.5–2 min to travel across each delay channel, which enables us to weigh each cell 10–12 times (depending on the number of sensors on the device) over the course of ~20 min (Fig. 1a, b and Supplementary Fig. 1). Cell MAR, defined as the net change in mass over time, is determined by calculating the slope of linear least squares fit as a function of time applied to a set of individual mass measurements from the same cell (Fig. 1b, c).

For MM, cells are measured in suspension following disassociation of clumped cells while maintaining the appropriate temperature and CO_2_ concentration for cell viability and growth. The sSMR measurement system has been described previously and in Supplementary Note 1 and Supplementary Fig. 5 ^26, 27^. In order to improve the reliability of measurement, dissociated single-cell suspensions are flowed through the device in media supplemented with 5 mM EDTA and 10 μg/mL PLL-PEG to prevent myeloma cells from sticking to the channel walls (Supplementary Note 2 and Supplementary Figs. 6 and 7). The resulting sSMR data are two-dimensional, capturing both MAR and the average single-cell mass over the duration of the measurement as independent biomarkers (Fig. 1d). By applying these measurements to the IL-6-dependent human MM cell line, ANBL-6, we can characterize the heterogeneity in mass and MAR across the population (Fig. 1d). Furthermore, the single-cell resolution of the MAR assay allows characterization of phenotypic subpopulations^18, 25^.

### MAR defines therapeutic response to proteasome inhibition

**Fig. 2:**
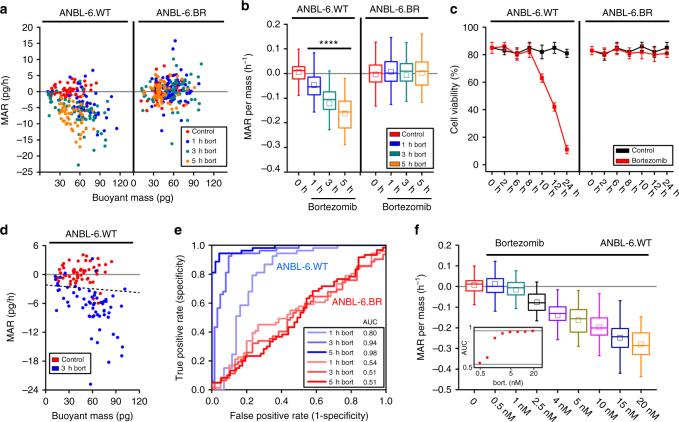
Human multiple myeloma ANBL-6 cells rapidly reduce MAR upon exposure to proteasome inhibition. **a** MAR vs. mass plot for bortezomib-sensitive (ANBL-6.WT) and bortezomib-resistant (ANBL-6.BR) cells exposed to 5 nM bortezomib (bort) with 1, 3, and 5 h-long treatment duration. **b** Same data as in **a** shown as MAR per mass box plot. Boxes represent the inter-quartile range and white squares are the average of all MAR measurements. Welch’s *t*-test has been used to calculate *p* values, comparing treatment groups to 0-h control. ****p < 10^−4^ in highlighted segments. All treatment groups for the ANBL-6.WT cells have *p* < 10^−5^, where for the bortezomib exposure from 1 to 5 h; *p*(control vs. 1-h. bort.) = 4.8 × 10^−5^, *p*(control vs. 3-h. bort.) = 5.3 × 10^−16^, and *p*(control vs. 5-h. bort.) = 4.7 × 10^−26^. From left to right, number of cells in MAR measurements; *n*(ANBL-6.WT) = 53, 43, 62, 47 and *n*(ANBL-6.BR) = 59, 72, 62, 57. **c** Cell viability by trypan blue showing the increase in the cell death for ANBL-6.WT upon exposure to bortezomib, while ANBL-6.BR cells are unaffected. Error bars represent three times the standard deviation with *n* = 3 for each condition. **d** Representative MAR vs. mass plot with overlay of an orthogonal vector (black dotted line), which designates the threshold resulting from LDA. **e** ROC curves of control (*t* = 0 h) and treatment (bortezomib) group for ANBL-6.WT and ANBL-6.BR cells at different treatment duration (*t* = 1, 3, 5 h). Cells, which are sensitive and resistant to drug therapy, are shown with blue and red lines, respectively. **f** MAR per mass plot for ANBL-6.WT cells exposed to different bortezomib concentrations, between 0.5 and 20 nM under 5 h-long treatment, showing the further reduction for larger bortezomib concentration. Figure inset shows the calculated AUC values for each bortezomib concentration, where AUC converges to 1 with increasing bortezomib concentration. Number of cells in MAR measurements from left to right; *n* = 53, 65, 58, 70, 61, 47, 52, 49, and 68

We first investigated the cellular response of MM cells to bortezomib, a proteasome inhibitor that is commonly used as frontline therapy^28–30^. Bortezomib leads to protein accumulation and cell death by impairing protein catabolism in the proteosome. We studied the impact of bortezomib on MAR and buoyant mass using wild-type ANBL-6 human MM cell line (ANBL-6.WT) and its bortezomib-resistant counterpart (ANBL-6.BR)^26, 27^. Treatment of ANBL-6 WT cells with bortezomib at a therapeutically relevant concentration of 5 nM for only 1 h significantly decreases MAR relative to baseline without altering the distribution of mass (Fig. 2a, b and Supplementary Fig. 2). The reduction of MAR becomes progressively more pronounced with longer durations of bortezomib exposure until all the cells have negative MARs, and the mass distribution begins to shift lower. In contrast, when the same conditions are applied to bortezomib-resistant ANBL-6.BR cells, no significant change is observed in either MAR, mass, or negative MAR fraction demonstrating that resistant cells maintain normal growth when subjected to inefficacious therapies (Fig. 2a, b and Supplementary Fig. 2). The same data can be represented on a single axis, where the MAR of each cell is normalized by the mass of that same cell (Fig. 2b). Mass is well characterized in clonal cell lines as a proxy for cell cycle position^24^, so by normalizing to cell mass we can account for size and cell cycle-dependent effects^18^. In comparison, bulk viability testing (Fig. 2c) required 10 h of bortezomib exposure to observe response, showing that reduction in MAR precedes loss of cell viability.

To demonstrate the robustness of our MAR measurements for classifying single-cell therapeutic sensitivity, we determined the receiver operating characteristics (ROC) after performing linear discriminate analysis (LDA) for each combination of treatment vs. control data sets. LDA projects the two-dimensional MAR and mass data onto a single axis that best distinguishes these two populations and defines a threshold for this classification (Fig. 2d). We then performed ROC curve analysis and calculated the area under the curve (AUC), which is a metric of the identification of each cell’s classification as sensitive or resistant to a drug^31^. For instance, a random classifier has an AUC = 0.5, while a perfect classifier has an AUC = 1. The AUC for all drug conditions tested on ANBL-6.BR cells is ~0.5, consistent with treated resistant cells being indistinguishable from untreated cells. In contrast, ROC curves for ANBL-6.WT cells show excellent resolution of treated and untreated groups as AUC converges to one for longer bortezomib exposure (Fig. 2e). To test whether increasing drug concentration allows for a better discrimination between treated vs. untreated cell populations, we exposed ANBL-6.WT cells to a range of bortezomib concentrations between 0.5 and 20 nM for 5 h and observed greater reduction in MAR at higher concentrations (Fig. 2f). As expected, AUC rapidly increases with concentration, approaching one for dosages at or above the therapeutically relevant 5 nM concentration.

### MAR defines response to combination therapy

**Fig. 3:**
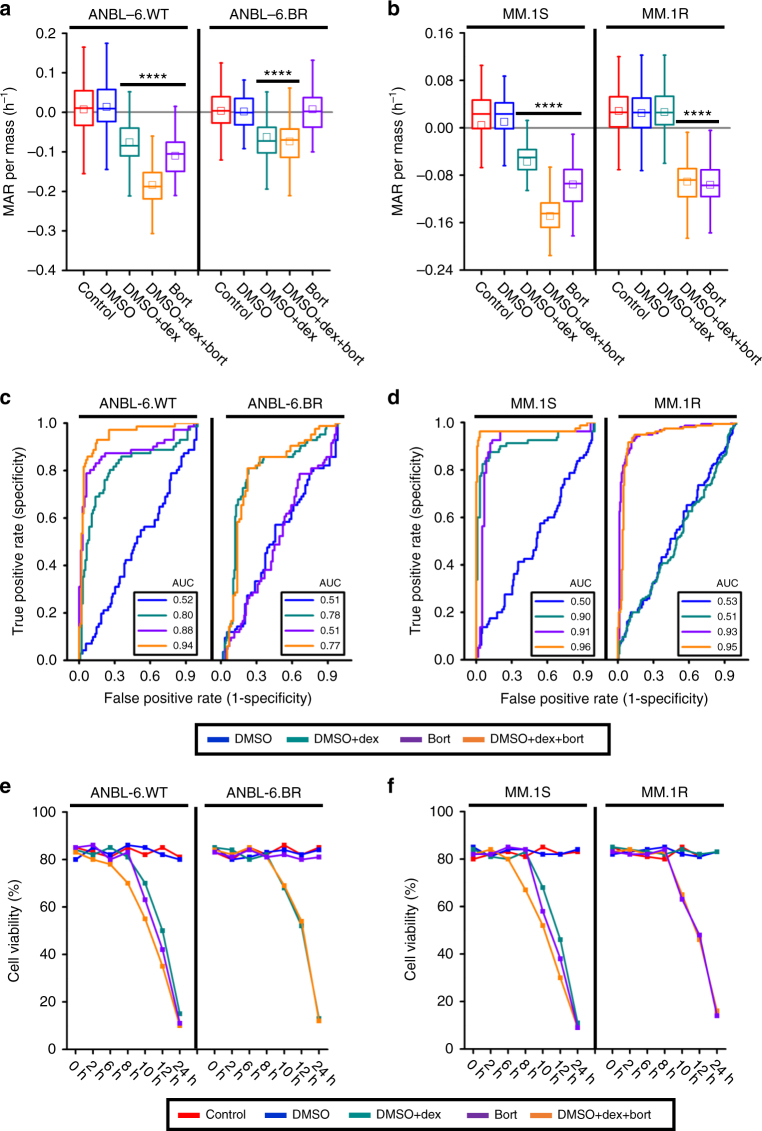
MAR defines drug sensitivity of human multiple myeloma ANBL-6 and MM.1 cells to bortezomib-dexamethasone combination. **a**, **b** MAR per mass of ANBL-6 and MM.1 cells treated in dimethyl sulfoxide (DMSO), 5 nM bortezomib, 200 nM dexamethasone and their combinations for 3 h. MAR per mass of ANBL-6.WT cells (bortezomib and dexamethasone sensitive) reduces upon the exposure to bortezomib and dexamethasone, while that of ANBL-6.BR cells (bortezomib resistant and dexamethasone sensitive) reduces only upon the exposure to treatment containing dexamethasone. MAR per mass of MM.1S cells (bortezomib and dexamethasone sensitive) reduces upon the exposure to bortezomib and dexamethasone, while that of MM.1R cells (dexamethasone resistant and bortezomib sensitive) reduces only upon the exposure to treatment containing bortezomib. Boxes represent the inter-quartile range and white squares are the average of all measurements. *p* values were calculated using Welch’s *t*-test, comparing treated cells to control (cells seeded only in culture media), and were Bonferroni corrected. ****p < 10^−4^ in highlighted segments. For ANBL-6.WT cells; *p*(DMSO vs. DMSO + dex.) = 3.2 × 10^−11^, *p*(control vs. bort.) = 1.5 × 10^−14^, *p*(DMSO vs. DMSO + dex. + bort.) = 3.7 × 10^−38^, for ANBL-6.BR cells; *p*(DMSO vs. DMSO + dex.) = 4.2 × 10^−6^, *p*(control vs. bort.) = 1, *p*(DMSO vs. DMSO + dex. + bort.) = 4.5 × 10^−4^, for MM.1S cells; *p*(DMSO vs. DMSO + dex.) = 7.6 × 10^−6^, *p*(control vs. bort.) = 6.3 × 10^−7^, *p*(DMSO vs. DMSO + dex. + bort.) = 2.3 × 10^−31^ and for MM.1 R cells, *p*(DMSO vs. DMSO + dex.) = 1, *p*(control vs. bort.) = 6.6 × 10^−52^, *p*(DMSO vs. DMSO + dex. + bort.) = 1.0 × 10^−44^. The number of cells in MAR measurements from left to right; *n*(ANBL-6.WT) = 61, 67, 63, 59, 64 and *n*(ANBL-6.BR) = 64, 59, 65, 68, 60 and *n*(MM.1S) = 60, 61, 64, 58, 59 and *n*(MM1.R) = 65, 61, 60, 64, 58. **c**, **d** ROC curves of control and treatment groups (DMSO, DMSO + dex, bort, DMSO + dex + bort) for ANBL-6 and MM.1 cells. **e**, **f** Cell viability analysis for ANBL-6 and MM.1 cells under different drug combinations

Next, we explored the concept of whether change in MAR can define response to combinations of agents, which is a treatment paradigm that has not been explored in previous studies of MAR. To fully validate this, we studied MAR response to a wider range of SOC single agents as well as to combinations of these agents used clinically. First, we evaluated the effect of dexamethasone and bortezomib alone and in combination in three human MM cell lines with variable dexamethasone and bortezomib sensitivity. This includes the ANBL-6.WT and ANBL-6.BR cell lines discussed above, the MM.1 cell line, which is either dexamethasone sensitive (MM.1S) or resistant (MM.1R), and the U266 cell line, which is sensitive to both agents. These cell lines were exposed to either 5 nM bortezomib or 200 nM dexamethasone alone or in combination for 3 h prior to measurement. As seen in Fig. 3a, the bortezomib-sensitive ANBL-6.WT cells show a significant reduction in MAR in all treatment groups compared to control. More importantly, the reduction in MAR is more pronounced in the drug combination, compared to the single agent dexamethasone or bortezomib. In contrast, we observe no additional magnitude of reduction in MAR following addition of bortezomib to dexamethasone in bortezomib-resistant ANBL-6.BR cells, These two observations confirm the ability of our MAR assay to selectively identify response to these two drugs. Analogously, both dexamethasone and bortezomib show a significant reduction in MAR as single agents in dexamethasone-sensitive MM.1S cell line, with the drug combination displaying more pronounced effect (Fig. 3b). This similarity holds for the dexamethasone-resistant MM.1R cell line where the reduction in MAR is the same for bortezomib alone or bortezomib in combination with dexamethasone; the dexamethasone-alone treatment group cannot be distinguished from controls. Results using U266 cell lines are analogous to those of the sensitive ANBL-6.WT and MM.1S cell lines (Supplementary Fig. 3).

The corresponding ROC curves for ANBL-6.WT and MM.1S show that the ability to resolve single cells between untreated and treated groups increases with combination therapy as compared to either therapy alone (Fig. 3c, d). The AUC converges toward one for drug combinations but remains constant in cell lines with resistance to either of the two agents alone. Serving as a good internal control, the bortezomib-resistant ANBL-6.BR cells treated with 5 nM bortezomib and dexamethasone-resistant MM.1R cells treated with 200 nM dexamethasone have AUC of ~0.5, a result indistinguishable from untreated control. Bulk viability responses show a reduction in viability for the drug combination that begins earlier in time compared to the monotherapies for ANBL-6.WT and MM.1S cells. In contrast, the timing of viability loss appears to be only due to the efficacious therapy in ANBL-6.BR and MM.1R cells treated with combination therapy (Fig. 3e, f). The timing of cell viability loss is correlated with the reduction in MAR following 3 h of drug exposure for all bortezomib and dexamethasone conditions tested, consistent with a progressive reduction of MAR up to a limit prior to loss of cell viability (Supplementary Note 3 and Supplementary Fig. 8).

**Fig. 4:**
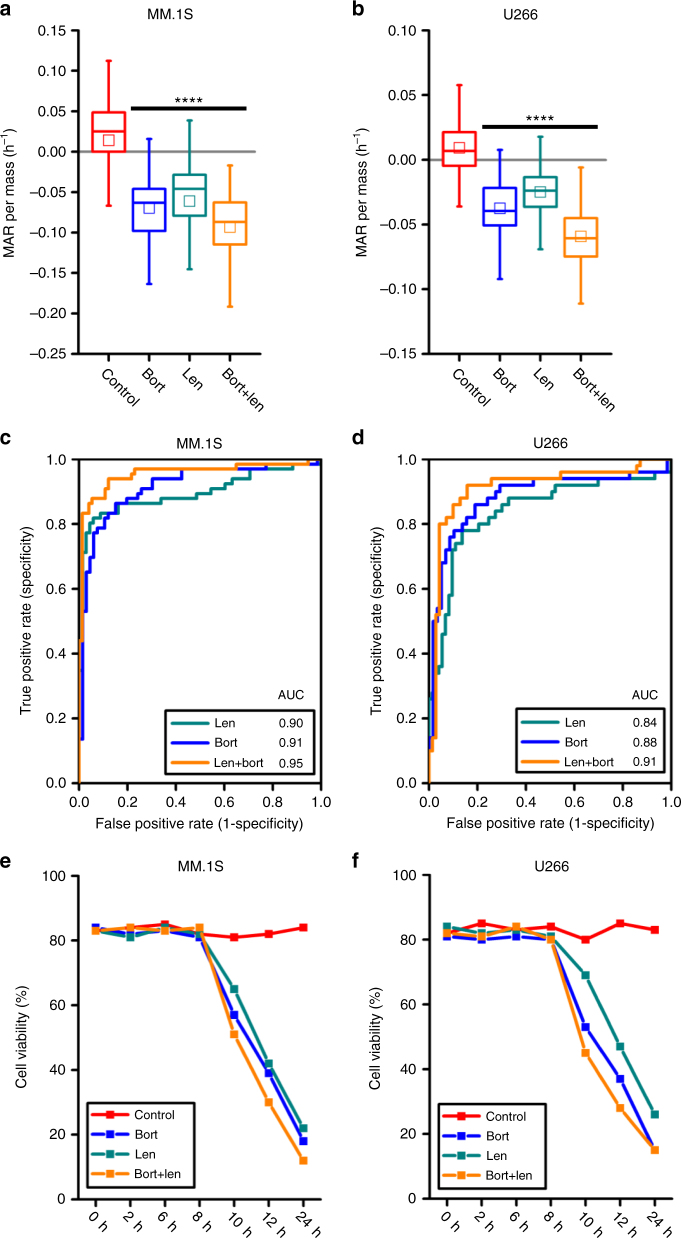
MAR defines drug sensitivity of human multiple myeloma MM.1S and U266 cells to lenalidomide therapy and its combination with bortezomib. **a**, **b** MAR per mass distributions of MM.1S and U266 cells treated in 5 nM bortezomib, 3 µM lenalidomide and their combinations for 3 h. MAR per mass of both cell lines reduces upon exposure to both drug treatment. Boxes represent the inter-quartile range and white squares the average of all measurements. *p* values were calculated using Welch’s *t*-test, comparing treated cells to the control (cells seeded only in culture media), and were Bonferroni corrected ****p < 10^−4^ in highlighted segments. For MM.1S cells; *p*(control vs. bort.) = 1.7 × 10^−9^, *p*(control vs. len.) = 1.1 × 10^−8^, *p*(control vs. bort. + len.) = 2.8 × 10^−16^ and for U266 cells; *p*(control vs. bort.) = 9.9 × 10^−11^, *p*(control vs. len.) = 5.9 × 10^−8^, *p*(control vs. bort. + len.) = 4.1 × 10^−15^. From left to right, number of cells in MAR measurement *n*(MM.1S) = 66, 66, 68, 74 and n(U266) = 50, 58, 73, 70. **c**, **d** ROC curves of control and treatment (bort, len, bort + len) data for MM.1S and U266 cells. **e**, **f** Cell viability analysis for MM.1S and U266 cells under different drug combinations

We next evaluated the effect on MAR of a highly efficacious cytotoxic and immunomodulatory drug, lenalidomide, alone and in combination with bortezomib using both bortezomib- and lenalidomide-sensitive U266 and MM.1S cell lines^32–34^. Similar to the aforementioned combination therapies, the combination of 5 nM bortezomib and 3 µM lenalidomide produces a greater reduction in MAR as compared to either drug alone (Fig. 4a, b). The corresponding ROC curves also demonstrate consistent behavior, with AUC values converging toward one when considering drugs in combination vs. monotherapies (Fig. 4c, d). Again, reductions in viability are first observed at 10 h of drug exposure, well after measured reductions in MAR at 3 h (Fig. 4e, f). Furthermore, in contrast to bortezomib and dexamethasone combination, viability measured at a 2-h interval was less correlated to the timing of viability loss (Supplementary Note 3 and Supplementary Fig. 8).

### MAR defines response to an investigational peptide-based inhibitors

**Fig. 5:**
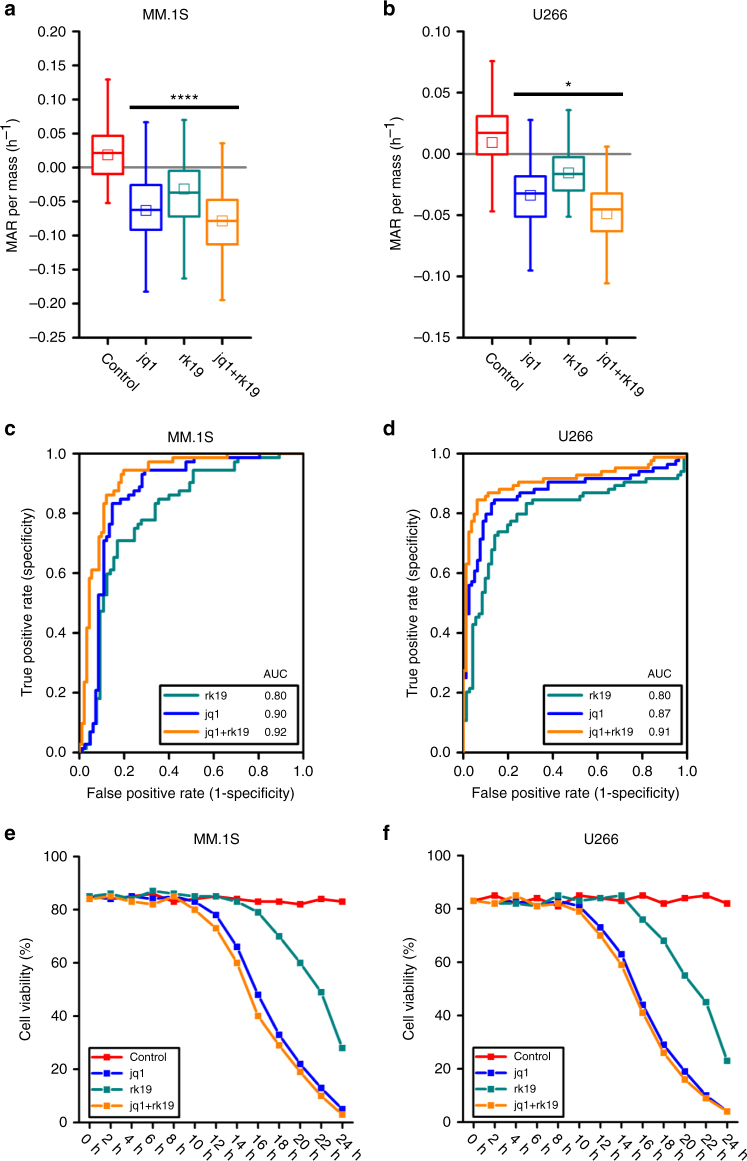
MAR defines sensitivity of human MM.1S and U266 cells to therapy jq1, rk19, and their combination. **a**, **b** MAR per mass distributions of MM.1S cells treated with 50 nM jq1, 20 µM rk19 and U266 cells treated with 200 nM jq1, 20 µM rk19 for 3 h. MAR per mass of both cell lines reduces upon exposure to both drug treatment. Boxes represent the inter-quartile range and white squares the average of all measurements. *p* values are calculated using Welch’s *t*-test, comparing treated cells to the control (only cell media), and were Bonferroni corrected. ****p < 0.0001 in highlighted segments. For MM.1S cells; *p*(control vs. jq1) = 6.4 × 10^−10^, *p*(control vs. rk19) = 3.9 × 10^−5^, *p*(control vs. jq1 + rk19) = 8.7 × 10^−21^ and *p < 0.05 in highlighted segments for U266 cells; *p*(control vs. jq1) = 1.3 × 10^−6^, *p*(control vs. rk19) = 0.013, *p*(control vs. jq1 + rk19) = 7.0 × 10^−11^. From left to right, number of cells in MAR measurement *n*(MM.1S) = 72, 82, 65, 91 and *n*(U266) = 84, 79, 71, 81. **c**, **d** ROC curves of control and treatment (jq1, rk19, jq1 + rk19) data for MM.1S and U266 cells. **e**, **f** Cell viability analysis for MM.1S and U266 cells under different drug combinations

Having confirmed the applicability of our assay to a subset of approved SOC agents in MM, we next evaluated its application in experimental setting using novel small molecule and peptide-based inhibitors. Recently, we have shown that the combined inhibition of BRD4 and E2F is effective at killing MM cells in vitro and in vivo in MM cell lines and primary MM cells^35^. BRD4 is inhibited via the BET bromodomain inhibitor JQ1, and E2F is inhibited using a modified, cell-penetrating polypeptide (rk19) with the ability to abrogate E2F1-DP1 heterodimerization and therefore suppress E2F activity. We tested MAR response in U266 and MM.1S cells following treatment with 50 nM JQ1 and 20 µM of rk19 blocking peptide alone or in combination. As seen in Fig. 5a, b, we observed reduction in MAR by both agents used alone and a more pronounced reduction when they are used in combination. The corresponding ROC curve also confirmed analogous changes to AUC with the administration of multiple drugs, where values converge to one for drugs in combination vs. monotherapies (Fig. 5c, d). Finally, the viability of the cell lines similarly shows that reduction in the viability begins earlier for drugs combination compared to single drug therapy. (Fig. 5e, f and Supplementary Note 3).

### Therapeutic sensitivity determined by MAR correlates with patient response

**Fig. 6:**
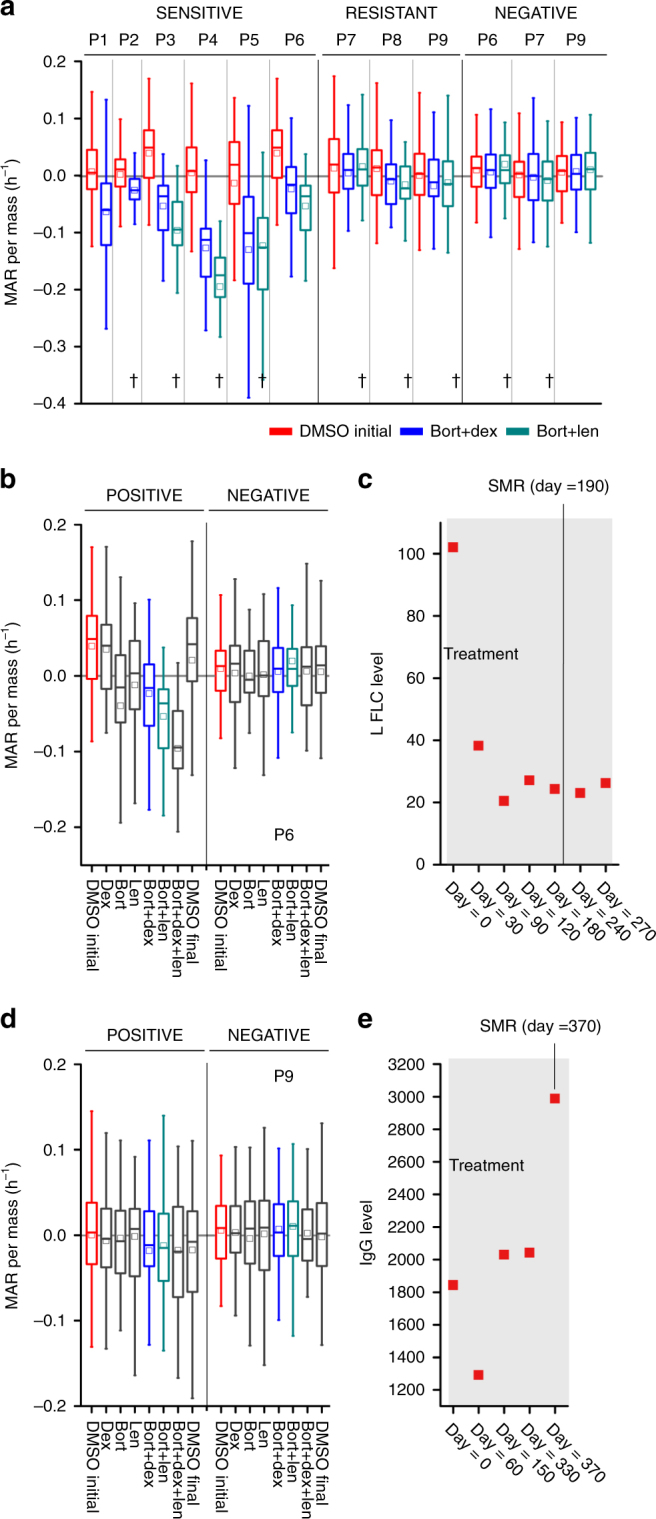
MAR defines therapeutic sensitivity in multiple myeloma patient samples. **a** MAR per mass of 12 samples from 9 patients is shown for control and treatment group under two drug combinations of 5 nM bortezomib, 200 nM dexamethasone, and 3 µM lenalidomide. Samples are divided into three groups, sensitive, resistant, and negative based on the clinical tests. Red denotes the initial control group response, and blue and green denote the treatment groups under dexamethasone + bortezomib and lenalidomide + bortezomib drug combinations with same drug concentrations as noted in. MAR per mass of sensitive group reduces upon exposure to drug treatments (*p* < 0.0056), while resistant (*p* > 0.47) and negative fraction (*p* = 1) show negligible variations. *p* values are calculated using Welch’s *t*-test, comparing treated cells to the control (only cell media), and were Bonferroni corrected. Full list of *p* values for **a**, can be found in Supplementary Note 4. Boxes represent the inter-quartile range and white squares the average of all measurements. From left to right, number of cells in MAR measurement *n* = 57, 60, 69, 56, 62, 64, 64, 65, 57, 63, 65, 67, 68, 64, 50, 62, 69, 71, 73, 64, 50, 62, 51, 54, 59, 53, 54, 49, 63, 59, 67, 61, 62. “†” denotes blinded tests. **b**, **d** MAR per mass of P6 and P9 is shown for control and treatment group under single, double, and triple drug combinations of 5 nM bortezomib, 200 nM dexamethasone, and 3 µM lenalidomide. **c**, **e** Clinical test results: L FLC and IgG levels for P6 and P9, determined at different time points before and after dexamethasone, bortezomib, and lenalidomide treatment

To confirm applicability of our assay in clinical setting and to validate MAR sensitivity determinations with actual clinical responses observed in the patients, we utilized purified primary myeloma cells from nine patients (P1-P9) isolated both before and following initiation of therapy (Fig. 6, Supplementary Note 4, Supplementary Figs. 9–17, and Supplementary Tables 1–13). Double-blinded methods were used for six of nine patients, (excluding P1, P2, and P7). For all patient samples, cells were measured on the SMR within the 24 h following selection by ficoll gradient and CD138+ magnetic-activated cell sorting from the whole bone marrow. In the case where samples were provided by outside institutions, cells were shipped at room temperature as whole bone marrow overnight. We evaluated change in MAR following drug exposure in purified CD138+ MM cells from all nine patients and the flow-through CD138-negative fractions (containing no MM cells) from patients P6, P7, and P9. In order to best characterize the behavior of primary samples, we dosed cells with single drugs and drugs in combination for 3 h prior to measurement on the SMR. This included 5 nM bortezomib, 200 nM dexamethasone, and 3 µM lenalidomide, or the combination of bortezomib with either dexamethasone or lenalidomide. For two patients, P6 and P9, the common clinical combination of all three drugs was also investigated. Following unblinding, patient samples were divided into sensitive and resistant groups based on evaluation of clinical tests and applying IMWG criteria (e.g., IgG or IgA, M-spike, Kappa FLC or L FLC levels; see “Methods” and Supplementary Note 4), as well as a third group for negative fractions.

MAR measurements of these samples correctly classified the response to drug combinations compared to clinical markers of response. For all nine patients and three negative fraction samples, we used a Bonferroni-corrected sensitivity threshold of *p* < 0.0056 vs. DMSO controls (Fig. 6 and Supplementary Note 4). Sensitive patient samples (P1-P6) showed greater reduction in MAR in response to combinations as compared to single therapies, mirroring response trends seen in cell lines (Fig. 6 and Supplementary Note 4). Furthermore, this trend held for the triple combination as compared to the combinations of two drugs in P6 (Fig. 6b, c and Supplementary Note 4). Across combinations of two drugs in sensitive patients, all samples treated showed reductions in MAR that are significantly lower than in resistant samples, beyond *p* < 0.0056 for all samples following Bonferroni correction (Fig. 6a and Supplementary Note 4). In comparison, in resistant samples (P7, P8, and P9), combinations of two drugs yield little to no change in MAR, with all corrected *p* values at *p* > 0.47 (Fig. 6a, d, e and Supplementary Note 4). MAR measurements of negative fractions (no MM cells) were also performed for the patient samples P6, P7, and P9. These negative fraction cells are expected to display no response to drug treatment given a lack of the targetable pathways, and consistently, samples treated with drugs alone or in combination show no reduction of MAR, with *p* = 1 for all conditions tested as compared to controls following Bonferroni correction (Fig. 6a and Supplementary Note 4). For sensitive patient samples, ROC curves of these single-cell data sets have an average AUC of 0.82 across all combination conditions tested. In comparison, AUC for combination conditions in resistant and negative samples were 0.52 in both cases, consistent with treated populations being indistinguishable from DMSO controls. The relative average AUCs of sensitive vs. resistant patient samples are consistent with MAR and mass having predictive power at the single-cell level (Supplementary Note 4). We also measured cell viability before and after each drug condition experiment and observed that cell viability showed negligible variations for both control and treatment conditions within the experiment duration for all patient samples (Supplementary Fig. 4).

## Discussion

This work demonstrates the capability of MAR measurements to functionally assess the therapeutic susceptibility of MM cells. Using human MM cell lines with known drug sensitivity, we confirmed the ability of MAR to correctly define response to SOC agents lenalidomide, bortezomib, and dexamethasone at the single-cell level. For these therapies, we show that MAR response assesses differential susceptibility of cells to drugs as monotherapies as well as in combination. Furthermore, MAR response was able to assess sensitivity to experimental MM therapies, including the BET inhibitor JQ1 and a peptide-based therapeutic targeting E2F/DP1 interaction, highlighting the potential of functional MAR measurements to assess therapeutic response in research setting.

**Fig. 7:**
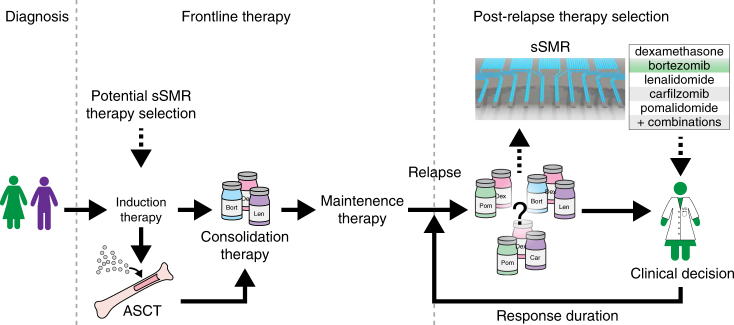
Schematic of treatment pipeline for multiple myeloma patients. Following diagnosis, patients undergo induction therapy (for example, combination of bortezomib, lenalidomide, and dexamethasone), followed by either consolidation therapy, or in eligible patients, autologous stem cell transplant (ASCT) with consolidation. This is followed by maintenance therapy. However, even with sustained maintenance therapy, almost all patients inevitably relapse. At the time of relapse, a clinical decision is made to choose from an array of therapeutic options, including many combination therapies. To inform this decision, patient history is considered (for example, prior therapies received) combined with the physician’s clinical experience (solid lines). Response duration varies, but eventually relapse occurs, and the same process repeats. Post-relapse drug selection is where the sSMR and assaying cell MAR response would be of greatest utility, allowing more precise clinical determination of therapeutic strategy by adding an important data point to the physician’s decision-making process (dotted lines). Results of the assay could inform which drug combinations are most likely to elicit complete response, as well as potentially being linked to other clinical outcomes such as progression-free survival. In addition to the post-relapse setting, MAR response could also help inform initial selection of induction therapy, especially with the growing list of available agents, to help maximize the probability of a complete response

Here we show that the MAR assay reveals drug sensitivity in primary myeloma cells across nine patients that were both sensitive or resistant to therapy. In cases of defined sensitivity, we detected a significant decrease in MAR following exposure to conventional single or combination therapies. Such reduction in MAR was not observed in resistant patients. Importantly, we have analyzed results both retrospectively (P3, P6, P7, P8, P9, Supplementary Note 4), where samples were collected after patients have received therapies, and prospectively (P1, P2, P4, P5, Supplementary Note 4), where we analyzed MAR response before patients received therapy. In both cases, MAR response was consistent with clinical outcomes, suggesting that this assay can be used to prospectively determine treatment decisions (Supplementary Note 4). The patient samples were randomly selected and represent a typical clinical scenario, where the majority of patients being treated for relapsed disease will remain sensitive to therapy, while a smaller fraction will present with resistant disease. Thus, our representative data suggests that the MAR assay has the potential to be used to select the best treatment options among both single and combination therapies in patients with relapsed disease, although additional SOC drugs must be tested for compatibility with the MAR assay (Fig. 7). This approach should also be evaluated for directing treatment choices in newly diagnosed patients, especially when using novel agents (Fig. 7).

Due to its unique characteristics, the MAR assay shows promise to make distinctive contributions to clinical practice as well as research. For clinical assessment, assaying the therapeutic sensitivity of primary MM samples ex vivo is more challenging than cell lines, since the amount of sample is often not sufficient for canonical bulk assays. In addition, cells do not proliferate without exogenous factors and viability declines rapidly once they are removed from the bone marrow niche. Here MAR assays were performed on primary samples as small as ~5 × 10^4^ cells split across seven conditions, and in previous research we assessed single-cell MAR response to therapeutics with as few as 1000 cells in 10–20 µL^18^. Reduction of MAR in myeloma cells occurs in less than 4 h following treatment, precedes loss of viability and does not require proliferation, making MAR measurements uniquely suited to working within the constraints of MM primary samples. Finally, because it is a single-cell approach, this assay also identifies cellular heterogeneity in response to each of the agent. In future research, it will be interesting to correlate this heterogeneity with the type and duration of clinical response achieved, or to study the effects of selected agents on non-malignant cells to predict toxicity.

In our assay, cells remain viable at the time of susceptibility measurement, and each single cell can be isolated downstream of the SMR^18^. For research applications, this capability combined with the ability to distinguish between sensitive and resistant populations in cell lines or primary samples, can enable heterogeneity in single-cell susceptibility phenotypes to be correlated with non-functional, genetic biomarkers, or other downstream orthogonal assays^18, 25, 36^. Driven by these correlations, molecular pathways associated with therapeutic resistance or other phenotypes could be identified and specifically targeted with combination strategies likely to be synergistic.

The MAR assay has its own set of challenges that appear on both sides of the therapeutic axis, involving cell state maintenance and capturing the cell-extrinsic factors related to drug response. As with other functional assays based on ex vivo treatment, many of the microenvironmental effects which influence in vivo drug response are excluded in ex vivo drug exposure. Bone marrow microenvironment cues significantly affect the survival of MM cells, and the removal of these signals for even less than 24 h could greatly affect drug response. Thus, it is likely that our drug sensitivity measurements, where ex vivo treatment is applied to isolated MM cells, primarily reflects cell intrinsic properties. Furthermore, therapies like lenalidomide and thalidomide have both direct cytotoxic effects and effects through immune modulation, which could not be observed in isolated MM cells treated exclusively ex vivo. Future studies should include the use of co-culture environments with stromal and immune cells prior to ex vivo treatment to assess impact of specific microenvironmental cues, or activation of certain cell-extrinsic responses.

## Methods

### Cell culture of the conventional cell lines

MM.1S, MM.1R, and U266 cells are maintained in suspension in RPMI-1640 media (Gibco, Ref#11875-093), supplemented with 10% FBS (Sigma-Aldrich, Ref#F4135), 0.02 M Hepes (Gibco, Ref#1X Antibiotic-Antimycotic (Gibco, Ref#15240-062), and kept in a 37 °C, 5% CO_2_, and humidified incubator. ANBL-6.WT and ANBL-6.BR cells are maintained in the same media supplemented also with 5 ng/mL of IL-6, while ANBL-6.BR cell media contains additional 2.5 nM bortezomib (Takeda), which is added to cell media every other passage. Cells are passaged every 4 days to 2 × 10^5^ cells/mL. The approximate cell concentration used in SMR experiments is 1.2 × 10^5^. All cell lines were kindly provided by sources previously described^8^, ATCC, or the German Collection of Microorganisms and Cell Cultures. All cell lines tested negative for mycoplasma.

For drug experiments, cells in 24-well plates are dosed with 5 nM bortezomib, 200 nM dexamethasone (Sigma-Aldrich), 3 µM lenalidomide (Celgene), 50 or 200 nM jq1 (kindly provided by Jun Qi), or 20 µM rk19. rk19 (Celtek Bioscience, LLC) has the sequence **A-A-V-A-L-L-P-A-V-L-L-A-L-L-A-P**-R-R-R-V-Y-D-A-L-N-V-L-M-A-M-N-I-I-S-K, where the N-terminal 16-a.a. sequence (bold) is a cell-permeable sequence^37^ and the C-terminal 19-a.a. sequence is the H2 fragment derived from the DEF box region in DP-1^38^. Peptide is purified by HPLC with purity greater than 90%. For SMR experiments including dexamethasone therapy, in addition to control tests, we also perform controls with the media containing DMSO (Sigma-Aldrich, Ref#D2438). Cells are kept drugged during the measurements, which lasts ~1.5 h. All conventional cell lines are suspended in their standard growth media. Only ANBL-6 cells grows in clumps, and a gentle pipetting is performed to dissociate cells from each other (Supplementary Note 2 and Supplementary Fig. 3). Replicate MAR assays were performed across ANBL-6 lines, but not for MM.1 and U266 lines. All bulk assays were performed in triplicate.

### Patient sample procurement and processing

Primary multiple myeloma specimens were collected from patients at the Dana-Farber Cancer Institute upon provision of informed consent under a tissue banking protocol (Dana-Farber Harvard Cancer Center (DF/HCC) protocol #07-150). The protocol has been approved by the DF/HCC institutional review board (IRB), and all relevant ethical regulations were followed. Bone marrow mononuclear cells and primary MM cells are isolated using Ficoll-Hypaque density gradient sedimentation from BM aspirates MM patients following informed consent and IRB (Dana-Farber Cancer Institute) approval. MM patient cells are separated from BM samples by antibody-mediated positive selection using anti-CD138 magnetic-activated cell separation microbeads (Miltenyi Biotech, Gladbach, Germany). For the ex vivo drug treatment, aliquots are treated with 5 nM bortezomib, 200 nM dexamethasone, and 3 µM lenalidomide and assessed using the serial SMR platform. Patient samples did not allow for replicates of individual conditions on the same samples due to samples size and other practical constraints.

### Workflow of serial SMR

After the sample preparation steps described above, cells in suspension are mixed with 8-micron polystyrene particles (Thermo Fisher Scientific, Ref#4208A). The particles provide a baseline for zero mass accumulation rate as well as a calibration reference for measuring absolute buoyant mass of the flowing cells. The sample is delivered to the device through PEEK tubing (IDEX-1577) that is connected to pressurized vials containing the sample and waste tubes. By controlling the pressures supplied to each vial, we set the flow rate of the cells such that they can flow through the device in 15–20 min. After the experiment, we analyze the data taken from each sensor for determining the MAR of the cells using a Hungarian-based matching algorithm that was discussed elsewhere^25^. The temperature of the device and the sample vial is kept at 37 °C by circulating heated water through the aluminum blocks holding the device and the sample vials. The temperature of the tubing between the sample vials and the device is also controlled using an extra layer of tubing around the PEEK tubing.

Before each MAR measurement, the SMR microfluidics is coated with 10 µM poly-L-lysine/polyethylene-glycol (PLL-PEG) (SuSoS AG, Ref#PLL(20)-g[3.5]- PEG(2)) in order to prevent cells from sticking to microchannel walls. In order to reduce cell clumping, we utilized cell media containing 5 mM ethylenediaminetetraacetic acid (EDTA) (Fluka Analytical, Ref#03690) and 10 uM PLL-PEG. See Supplementary Note 2 for the effect of cell stickiness on time delay of cell travel between two sensors. Between runs, cleaning protocols are performed in order to remove any organic residue sequentially using filtered solutions of 0.25% trypsin or 10% bleach and then water followed by 5% Micro-90.

Comparisons between resultant single-cell data are performed by various statistical methods. Welch’s *t*-test was commonly applied to compare conditions where distributions of single-cell measurements are normal with similar variance. All *p* values were Bonferroni corrected.

### Serial SMR devices

All the samples investigated in this work are analyzed in devices that are fabricated by CEA-LETI on 8-inch silicon wafers using previously described microfabrication methods^22, 39^. Detailed information on the design of the devices and the measurement platform were given elsewhere^25^. Changes and improvements are described in the Supplementary Note 1.

### Code availability

The code used to generate the findings of this study are previously described in Cermak, N. et al. *Nat. Biotechnology* (2016)^25^.

### Data availability

The data that support the findings of this study are available from the authors on reasonable request.

## Electronic supplementary material

Supplementary Information

Peer Review File
