# Supplementary material for: Determining therapeutic susceptibility in multiple myeloma by single-cell mass accumulation
Source: Nat Commun. 2017 Nov 20;8:1613. doi: 10.1038/s41467-017-01593-2 (PMC5694762; doi:10.1038/s41467-017-01593-2)
Supplement: Supplementary file 1 — Supplementary Information [file 41467_2017_1593_MOESM1_ESM.pdf]

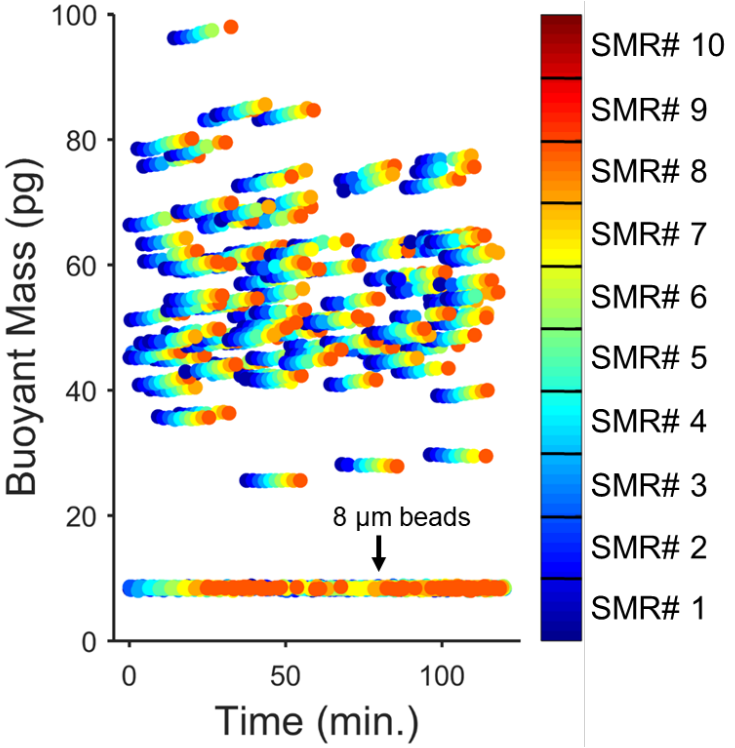

Supplementary Figure 1 Cell mass (L1210 hematopoietic cell line) is calculated from frequency shifts using 8 μm polystyrene calibration beads with known mass. Blue (SMR #1) to red (SMR #10) dots correspond to the mass of the same cell sequentially measured with 10 different SMRs.

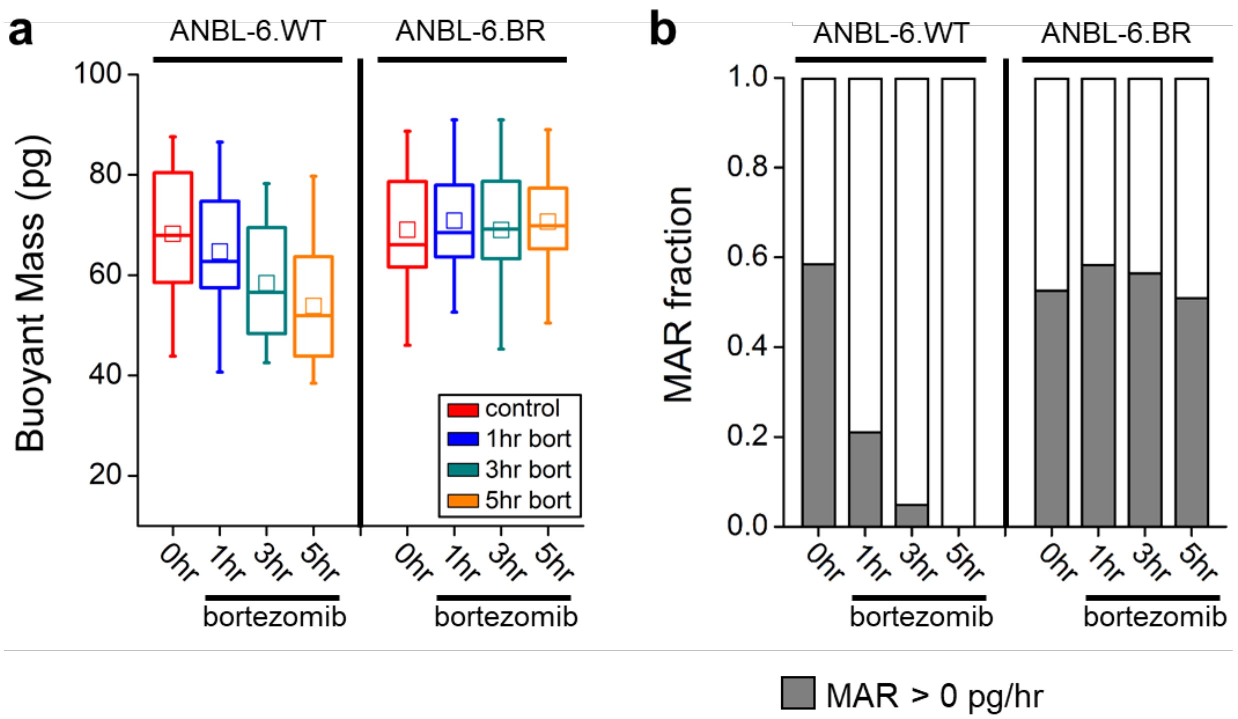

Supplementary Figure 2 (**a**) Same data as in Fig. 2a shown as mass box-plot. Boxes represent the inter-quartile range and white squares are the average of all mass measurements. (**b**) Same data as in Fig. 2a in the main text, showing that the fraction of ANBL-6.WT cells with negative MAR increases upon exposure to bortezomib but remains unaffected in ANBL-6.BR cells.

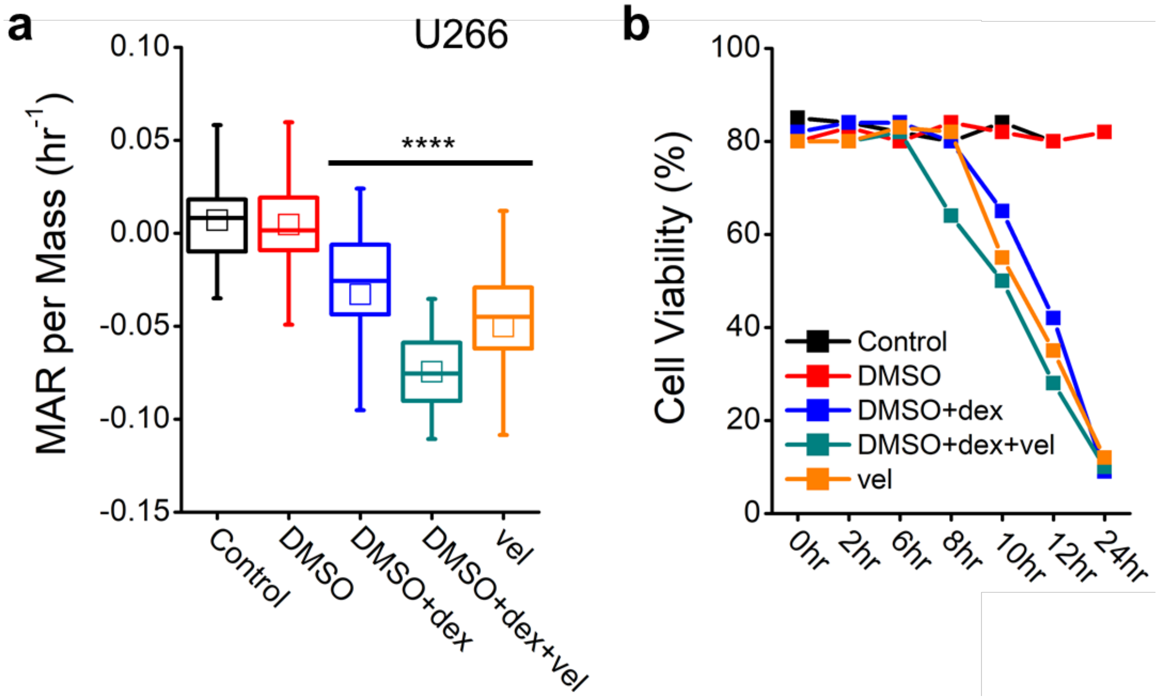

Supplementary Figure 3 MAR predicts drug sensitivity of human multiple myeloma U266 cells to bortezomib-dexamethasone combination. (**a**) MAR per mass of U266 cells treated in dimethyl sulfoxide (DMSO), 5 nM bortezomib, 200 nM dexamethasone and their combinations. MAR per mass of U266 reduces upon the exposure to bortezomib and dexamethasone. Boxes represent the inter-quartile range and white squares are the average of all measurements. p-values were calculated using Welch's t-test, comparing treated cells to control (cells seeded only in culture media), and were Bonferroni corrected. **** $p<10^{-4}$ in highlighted segments. p(DMSO vs. DMSO+dex.) = 9.7×10^{-5}, p(control vs. bort.) = 8.7×10^{-9}, p(DMSO vs. DMSO+dex.+bort.) = 6.6×10^{-31}. The number of cells in MAR measurements from left to right; n = 56, 52, 51, 62, 60. (**b**) Cell viability analysis for U266 cells under different drug combinations.

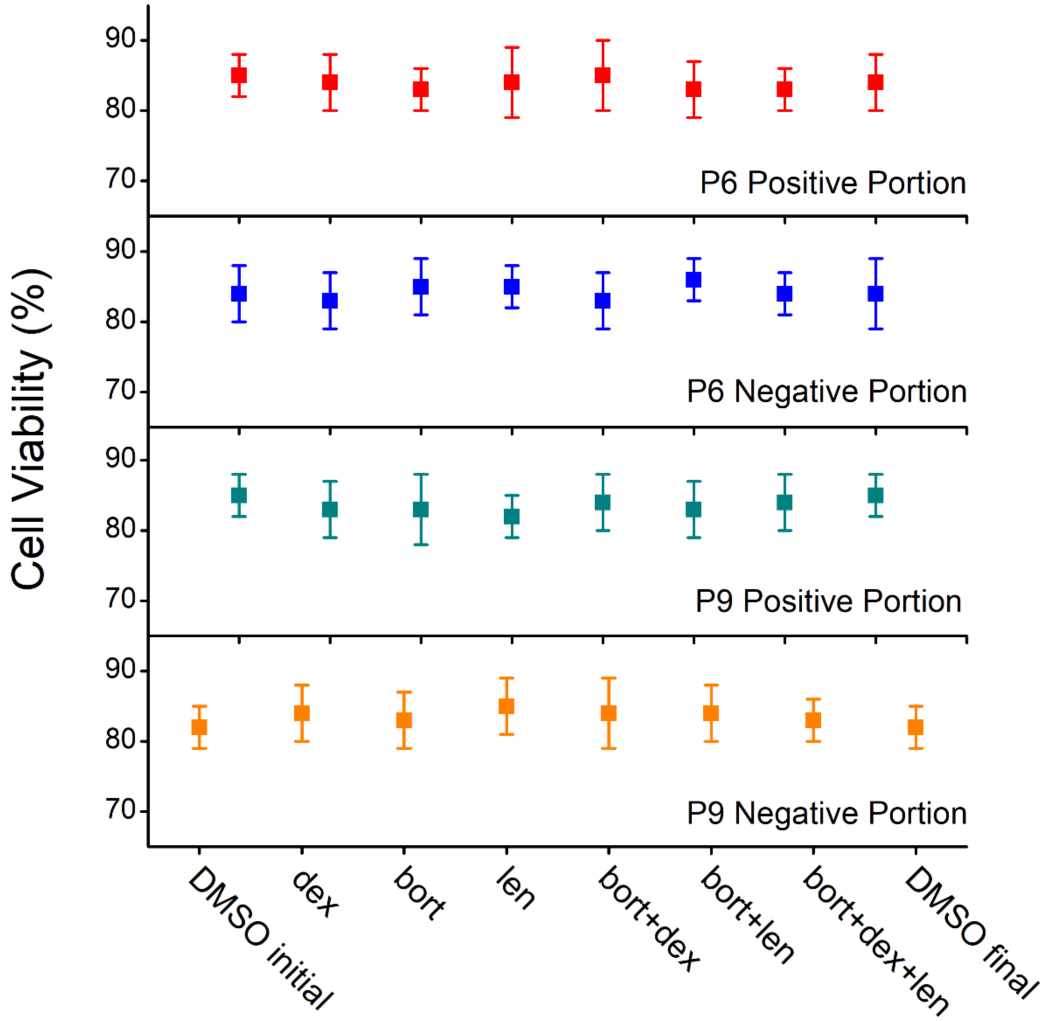

Supplementary Figure 4 Cell viability measured by trypan blue for all conditions from primary sample P6 and P9, for positive and negative portions. Measurements were taken before dosing, and after completion of MAR measurement for all experimental groups. Here, data is shown for the mean of 3 measurements and error bar is the three times the standard deviation.

SUPPLEMENTARY NOTE 1: Experimental system setup

Different from the serial SMR (sSMR) systems previously developed and described by Cermak et al.[1], here we have utilized sSMRs with piezoresistive sensors. Dedicated piezoresistors (15-20 kohms) for each SMR enable us to electrically measure motion of the cantilever mass sensors instead of using bulky optical components[2-4]. Removal of the optical components for measuring the motion of the sensors simplifies the system and increases its robustness and its ease of use. We used readout circuitry utilizing a tunable Wheatstone bridge with two channels. Each channel carries the signals for five to six piezoresistors combined. The signal in the Wheatstone bridge is amplified by a high-speed difference amplifier (AD8130) followed by a 4th order band-pass filter. The resulting signal is amplified and supplied to the analog-to-digital convertors of the FPGA controlling the cantilevers. The technology about simultaneously controlling multiple cantilevers was previously introduced by Olcum et al.[5] The drive signals of each SMR is summed in the FPGA and amplified by a high-current amplifier (LT1210) before driving a single piezoceramic placed underneath the device.

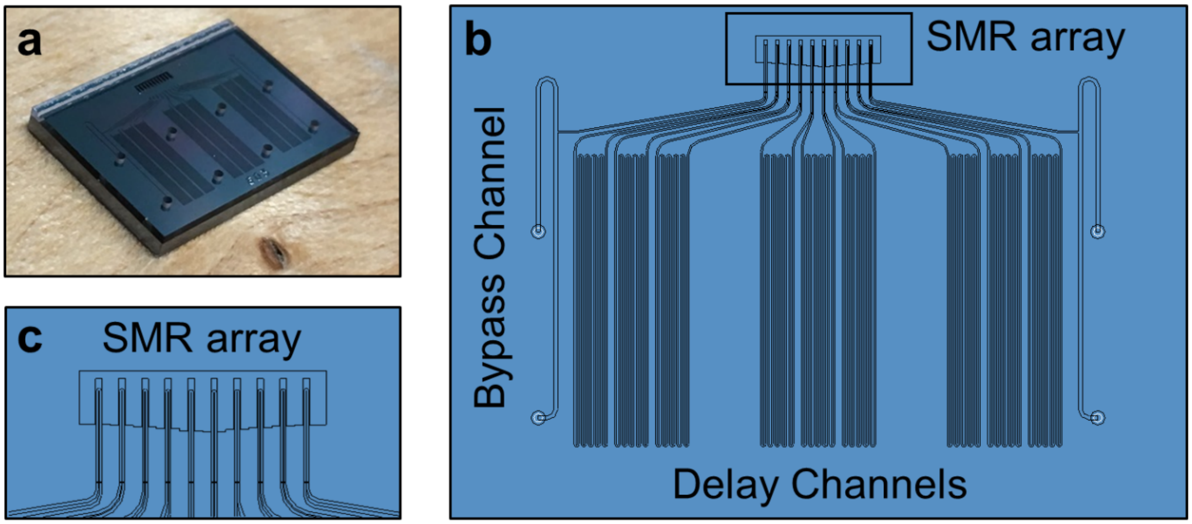

Supplementary Figure 5 SMR platform design. (**a**) The photograph of an SMR device and (**b,c**) rendering of a serial SMR device, showing bypass and delay channels with cantilevers magnified in the figure inset.

SUPPLEMENTARY NOTE 2: Measurement and operational considerations due to cell stickiness

Following resuspension or disassociation of cultures to a single-cell suspension we observed that myeloma cells would begin to adhere to the silicon surface of the device after only 30 minutes of flow through the device. This trend would eventually result in clogging in the microfluidic channels after ~1.5-2 hours. Additionally, for ANBL-6 cells which grow in clumps, we were concerned that this trend may suggest that cell clumps may aggregate upstream of measurement. While visual observation showed no evidence of cell clumps being measured, this trend suggested that events measured in the SMR may not just be single-cell events but could include clumps of 2 or more cells. To rule this out by an additional method, we compared the CVs of cell volume distributions measured by coulter counter from populations immediately following dissociation of after the 2 hr experiment. We found that the two were the same, consistent with the single-cell distribution being over this interval (**Panel a and b immediately below**).

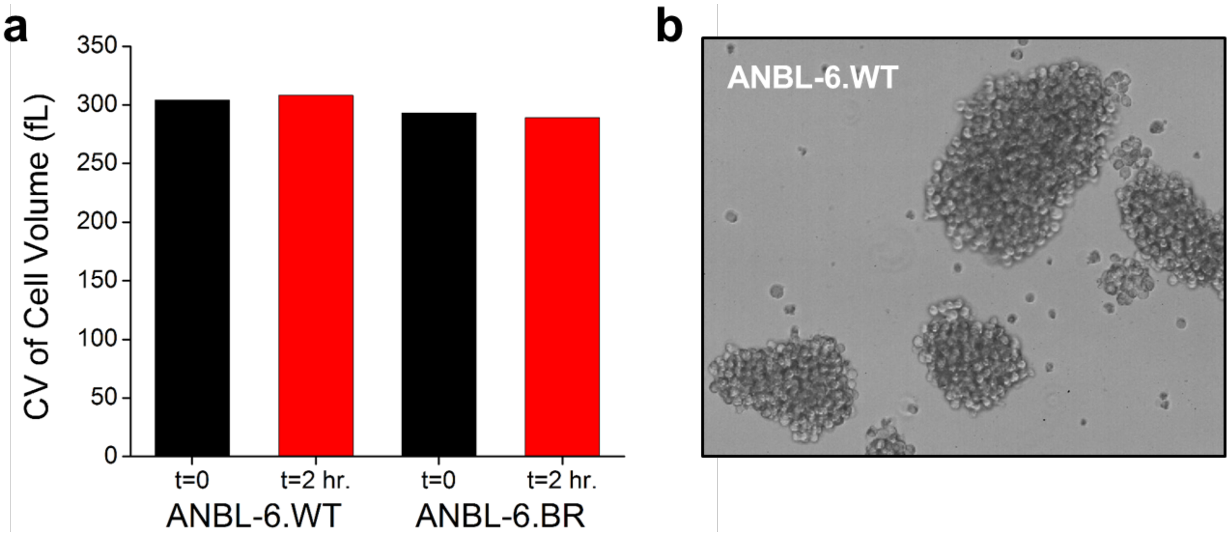

Supplementary Figure 6 (**a**) CV of the volume distribution of ANBL-6.WT cells over a 2 hour-long period. This distribution demonstrates that mechanical disassociation by gentle pipetting and filtering out large aggregates right before the MAR measurements ensures that only single-cells are measured. (**b**) ANBL-6.WT cells growing in clumps.

In addition, to qualitatively assess that the cells were sticking to channel walls, we compared the travel time of cells between the first two cantilevers and the last two cantilevers of the array, normalizing to the travel time of the calibrations beads in the same media solution. We drew comparisons between the well characterized L1210 hematopoietic cell line, a particle-like murine lymphocytic leukemia cell line known to not interact with channel walls, and multiple myeloma cell lines ANBL-6, MM.1 and U266 cells, and patient samples. The average of the normalized cell travel times in the first delay channel (between SMR#1 and SMR #2) and the last delay channel (between SMR #9 and SMR#10) were: L1210 - 0.67 and 0.67; MM cell lines - 0.79 and 0.81, and MM patient samples - 0.81 and 0.83. A higher average travel time for the multiple myeloma samples as compared to L1210

cells is consistent with multiple myeloma cells sticking more frequently to channel walls. The variation of the cell travel time for each cell type also shows similar trends, i.e, for L1210: 0.020 and 0.020, for multiple myeloma cell lines: 0.023 and 0.024, and multiple myeloma patient samples: 0.024 and 0.024.

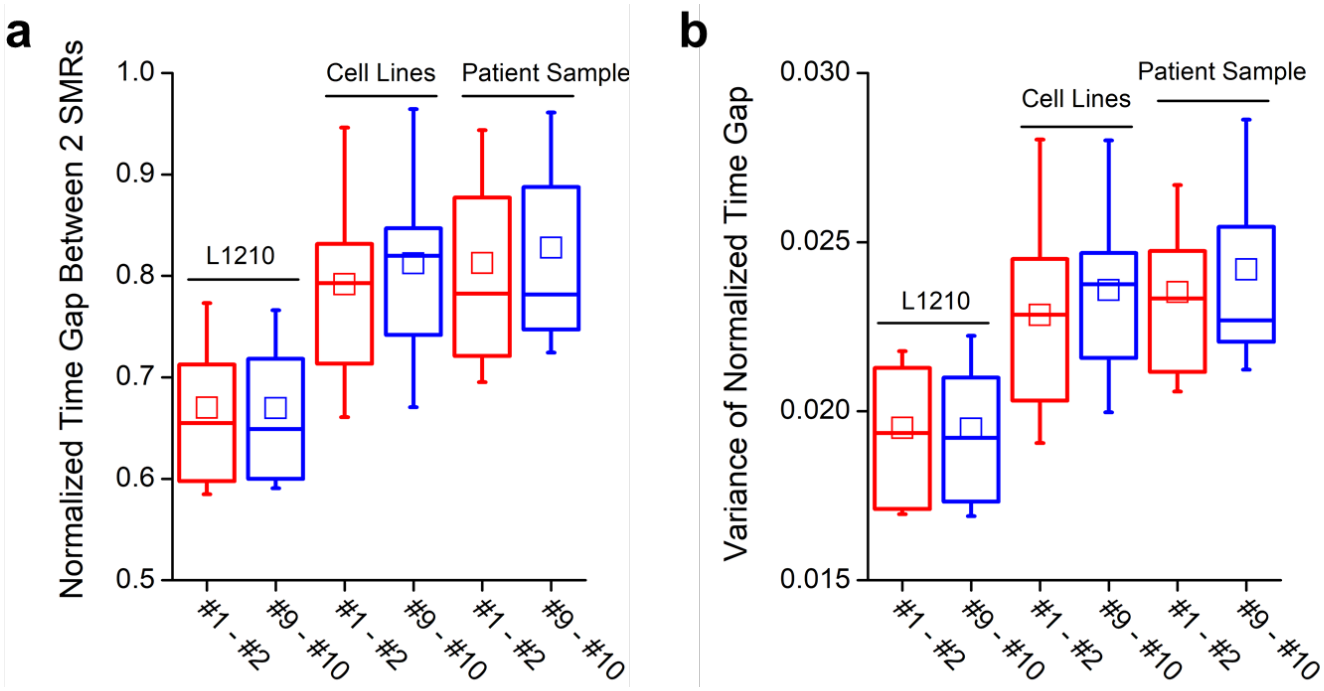

Supplementary Figure 7 (**a**) Normalized travel time and (**b**) variance of normalized travel time between the first two and the last two cantilevers (red and blue box-plots, respectively). Boxes represent the inter-quartile range and white squares are the average of all measurements. Number of experiments for each calculation; n = 8, 23, 8.

SUPPLEMENTARY NOTE 3: Correlation between MAR & timing of cell viability loss

Panel a shows the correlation between the change in the MAR and the change in cell viability for ANBL-6 and MM.1 subpopulations under the treatment with dexamethasone, bortezomib or their combination. Here, the viability is determined by taking the integral of the area (grey area in **Panel b**) under the viability curve as measured by trypan blue exclusion (red dots in **Panel b** are the average measured viability of the cells for triplicate measurements). The x-axis in **Panel a** is calculated by normalizing the integral of each treatment group to that of the control. The figure demonstrates the excellent correlation between the timing of initial viability loss and the reduction in the MAR for different drug treatment. **Panel c and d** show the integral ratio for MM.1S and U266 cell lines under the single and combination therapies of lenalidomide/bortezomib, and JQ1/rk19, respectively.

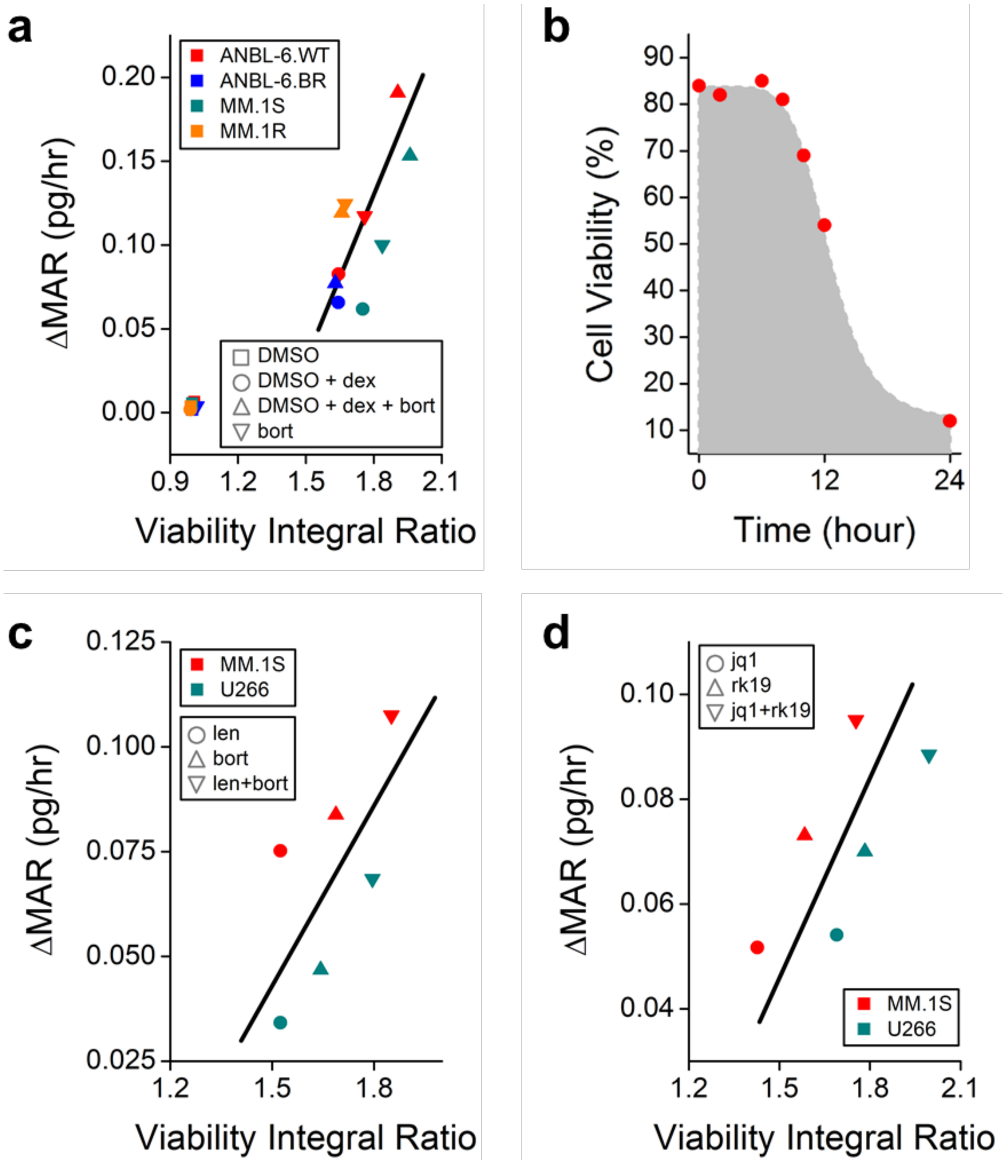

Supplementary Figure 8 Reduction in the cell viability vs. that of MAR. Color codes correspond to cell subpopulations and the shapes correspond to drug treatments: (**a**) dexamethasone + bortezomib, (**c**) lenalidomide + bortezomib, (**d**) JQ1 + rk19 treatments. (**b**) Cell viability (red dots) and the integral of the area under viability curve (grey area).

SUPPLEMENTARY NOTE 4: MAR results and clinical data for patient samples

In this section, we show the normalized MAR of the multiple myeloma patient samples for different drug treatments, the Bonferroni corrected statistics for each comparison made (p-values in the parentheses are before the correction), and the AUC value as an indicator of how distinguishable treatment conditions are from the control. We also show the clinical results (e.g. IgG or Kappa FLC) taken regularly over the course of patient treatment and disease monitoring, which was used in conjunction with other clinical data to determine the clinical sensitivity of each patient sample to the SOC therapies measured. For all patient data below, the figures on the left are boxplots of MAR data, and on the right show the IgG or FLC levels measured over time. The treatment duration, from start to finish, is highlighted with the green box and the date of sample isolation and MAR measurement is donated with "SMR". In addition, we have included a summary table of patient status, treatment received and IMWG classified response for each patient immediately below.

Patient #	Patient Status at MAR	Treatment	IMWG* Response
1	Newly diagnosed	RVD	VGPR
2	Post therapy	VD	PR
3**	Post therapy	RD	NR
4	Newly diagnosed	RVD	nCR
5	Post therapy	VD	PR
6	Post therapy	RD	nCR
7	Post therapy	RVD	PD
8	Post therapy	RVD	PD
9	Post therapy	RVD	PD

Supplementary Table 1 IMWG classified response for each patient. Treatments abbreviated with single-letter designations where R is lenalidomide, V is bortezomib, and D is dexamethasone.
* VGPR, very good partial response; PR, partial response; PD, progressive disease; NR, no response; and nCR, near complete response. ** Following RD therapy, patient received bortezomib, pomalidomide and dexamethasone and achieved CR with in 2 cycles (60 days)

PATIENT SAMPLE 1

Patient 1 (P1) was classified as "sensitive" to dexamethasone, bortezomib and lenalidomide. The drop in the IgG level was observed over the 70 day-long treatment interval with dexamethasone, bortezomib and lenalidomide. MAR measurement was performed 15 days before the beginning of the treatment.

	DEX	BORT	BORT+DEX	FINAL
**p-value**	1 (0.54)	0.038 (0.0094)	8.5×10^{-5} (2.1×10^{-5})	1 (0.82)
**AUC**	0.60	0.66	0.78	0.52

Supplementary Table 2 Table of p and AUC values for Patient 1 treatment groups. Values calculated using initial control for comparison to each condition. p-values were determined by Welsh's t-test and Bonferroni corrected. Uncorrected p-value in parentheses.

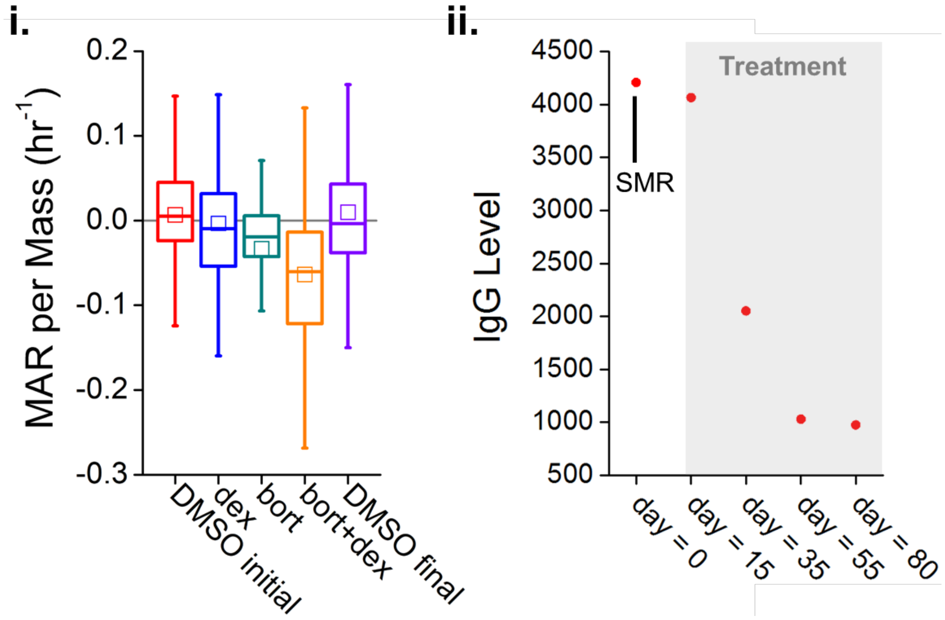

Supplementary Figure 9 (**i**) Normalized MAR for control and treatment groups. Boxes represent the inter-quartile range and white squares are the average of all measurements. From left to right, number of cells in MAR measurement n = 57, 63, 62, 60, 60. (**ii**) IgG level taken at different time points before and after the treatment. Treatment interval denoted with grey box, timing of SMR measurement denoted with "SMR".

PATIENT SAMPLE 2

Patient 2 (P2) was classified as "sensitive" for dexamethasone and bortezomib and "partial sensitive" for lenalidomide. The drop in the IgG level was observed within a 40 day-long period of treatment time with bortezomib + dexamethasone. The MAR measurement was performed 20 days before the beginning of the treatment.

	DEX	BORT	BORT+DEX	FINAL
**p value**	1<br>(0.43)	1<br>(0.28)	0.0056<br>(0.0014)	1<br>(0.94)
**AUC**	0.57	0.64	0.72	0.52

Supplementary Table 3 Table of p and AUC values for Patient 2 treatment groups. Values calculated using initial control for comparison to each condition. p-values were determined by Welsh's t-test and Bonferroni corrected. Uncorrected p-value in parentheses.

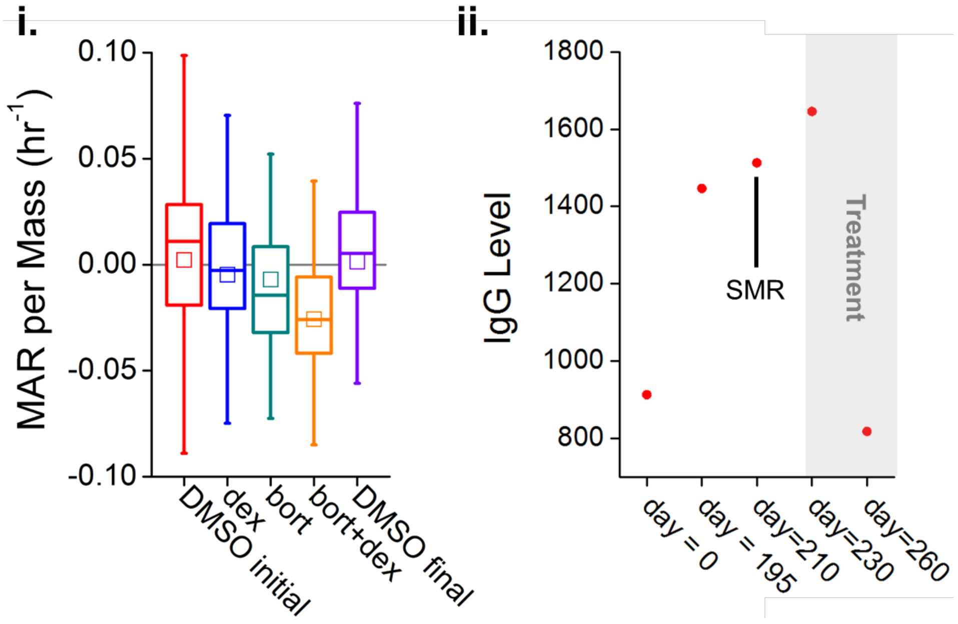

Supplementary Figure 10 (**i**) Normalized MAR for control and treatment groups. Boxes represent the inter-quartile range and white squares are the average of all measurements. From left to right, number of cells in MAR measurement n = 69, 58, 66, 56, 69. (**ii**) IgG level taken at different time points before and after the treatment.

PATIENT SAMPLE 3

Patient 3 (P3) was classified as "partial sensitive" for dexamethasone and lenalidomide and "naïve" for bortezomib. The response to lenalidomide + dexamethasone treatment was classified as no response based on M-spike data (**Supplementary Fig. 8c-ii**). However, patient responded robustly once transferred to bortezomib-based combination therapy (**Supplementary Fig. 8c-iii**).

	DEX	BORT	LEN	BORT+DEX	BORT+LEN	FINAL
**p value**	1 (0.45)	0.0014 (2.27×10^{-4})	0.020 (0.0033)	1.2×10^{-9} (2.0×10^{-10})	1.3×10^{-18} (2.2×10^{-19})	1 (0.48)
**AUC**	0.57	0.69	0.66	0.85	0.88	0.51

Supplementary Table 4 Table of p and AUC values for Patient 3 treatment groups. Values calculated using initial control for comparison to each condition. p-values were determined by Welsh's t-test and Bonferroni corrected. Uncorrected p-value in parentheses.

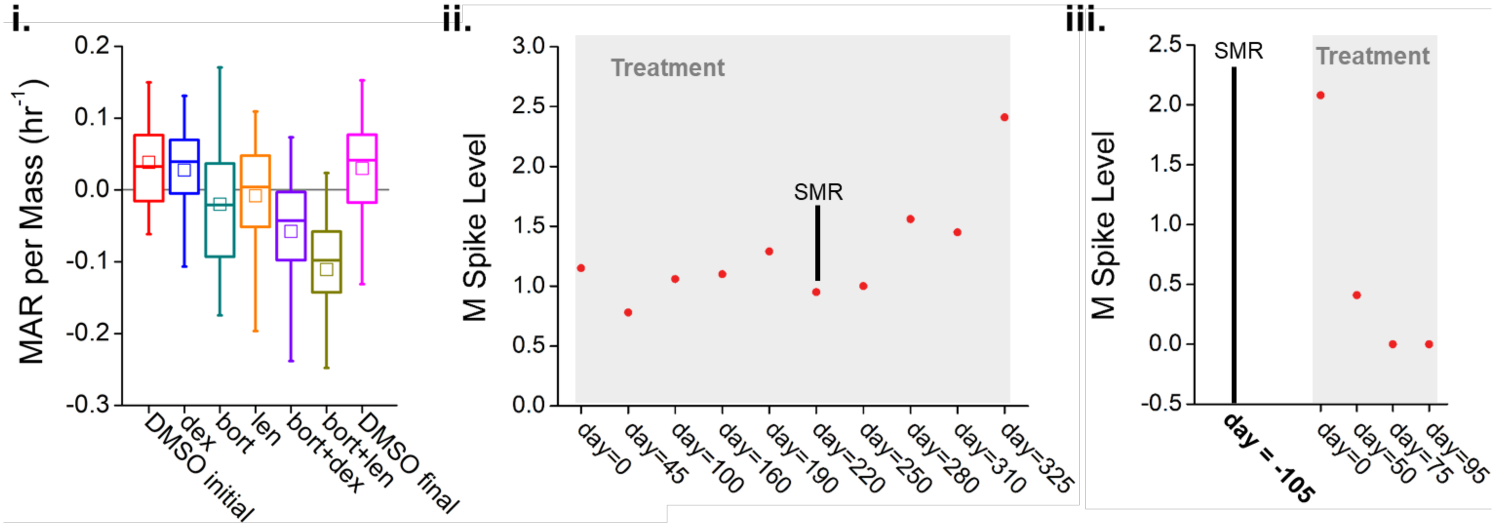

Supplementary Figure 11 (**i**) Normalized MAR for control and treatment groups. Boxes represent the inter-quartile range and white squares are the average of all measurements. From left to right, number of cells in MAR measurement n = 62, 52, 53, 54, 64, 64, 68. M-Spike level taken at different time points before and after treatment with (**ii**) lenalidomide + dexamethasone and (**iii**) bortezomib + pomalidomide + dexamethasone treatments.

PATIENT SAMPLE 4

Patient 4 (P4) was classified as "sensitive" for dexamethasone, lenalidomide and bortezomib. The drop in the Kappa FLC level was observed 15 days after the treatment start date with bortezomib + dexamethasone + lenalidomide. MAR measurement was performed 15 days before the beginning of the treatment.

	DEX	BORT	LEN	BORT+DEX	BORT+LEN	FINAL
**p value**	0.15 (0.026)	8.2×10^{-10} (1.3×10^{-10})	0.013 (0.0021)	6.7×10^{-15} (1.1×10^{-15})	7.5×10^{-25} (1.3×10^{-25})	1 (0.81)
**AUC**	0.56	0.75	0.71	0.87	0.93	0.55

Supplementary Table 5 Table of p and AUC values for Patient 4 treatment groups. Values calculated using initial control for comparison to each condition. p-values were determined by Welsh's t-test and Bonferroni corrected. Uncorrected p-value in parentheses.

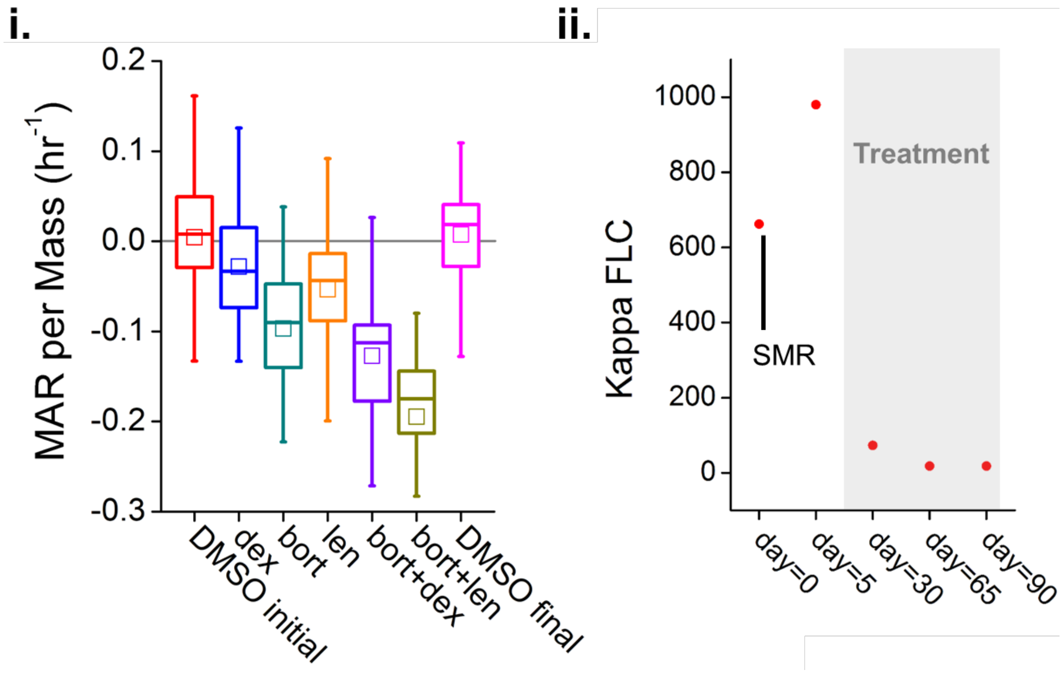

Supplementary Figure 12 **(i)** Normalized MAR for control and treatment groups. Boxes represent the inter-quartile range and white squares are the average of all measurements. From left to right, number of cells in MAR measurement n = 65, 52, 59, 67, 57, 63, 66.

PATIENT SAMPLE 5

Patient 5 (P5) was classified as "partial sensitive" for lenalidomide and "sensitive" for dexamethasone and bortezomib. The drop in the IgG level was observed within a 20 day-long period of treatment time with bortezomib + dexamethasone. The MAR measurement was performed 15 days before the beginning of the treatment.

	DEX	BORT	LEN	BORT+DEX	BORT+LEN	FINAL
**p value**	1 (0.28)	0.039 (0.0065)	0.62 (0.10)	9.7×10^{-5} (1.6×10^{-5})	1.2×10^{-5} (2.0×10^{-6})	1 (0.62)
**AUC**	0.64	0.72	0.68	0.80	0.84	0.53

Supplementary Table 6 Table of p and AUC values for Patient 1 treatment groups. Values calculated using initial control for comparison to each condition. p-values were determined by Welsh's t-test and Bonferroni corrected. Uncorrected p-value in parentheses.

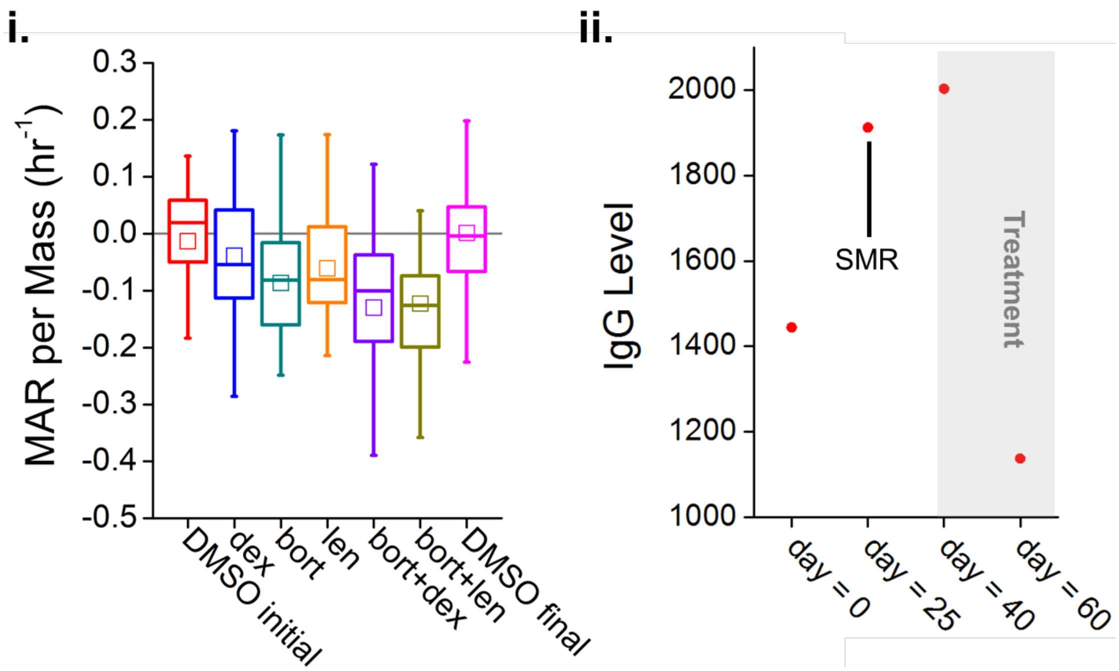

Supplementary Figure 13 (**i**) Normalized MAR for control and treatment groups. Boxes represent the inter-quartile range and white squares are the average of all measurements. From left to right, number of cells in MAR measurement n = 65, 60, 61, 57, 67, 68, 68. (**ii**) IgG level taken at different time points before and after the treatment.

PATIENT SAMPLE 6

Patient 6 (P6) was classified as “sensitive” for dexamethasone and lenalidomide, and “naïve” for bortezomib. The drop in the L FLC level was observed within a 30 day-long period of treatment time with lenalidomide + dexamethasone. The MAR measurement was performed 190 days after the beginning of the treatment.

	DEX	BORT	LEN	BORT+DEX	BORT+LEN	BORT+DEX+LEN	FINAL
**p value**	1<br>(0.23)	2.0×10^{-4}<br>(2.9×10^{-5})	0.0025<br>3.5×10^{-4}	2.0×10^{-5}<br>(2.9×10^{-6})	5.8×10^{-12}<br>(8.3×10^{-13})	6.4×10^{-19}<br>(9.1×10^{-20})	1<br>(0.23)
**AUC**	0.56	0.75	0.71	0.78	0.87	0.93	0.55

Supplementary Table 7 Table of p and AUC values for Patient 6, CD138+ fraction treatment groups. Values calculated using initial control for comparison to each condition. p-values determined by Welsh’s t-test and Bonferroni corrected. Uncorrected p-value in parentheses.

	DEX	BORT	LEN	BORT+DEX	BORT+LEN	BORT+DEX+LEN	FINAL
**p value**	1<br>(0.62)	1<br>(0.37)	1<br>(0.51)	1<br>(0.76)	1<br>(0.45)	1<br>(0.77)	1<br>(0.72)
**AUC**	0.50	0.55	0.52	0.50	0.52	0.52	0.51

Supplementary Table 8 Table of p and AUC values for Patient 1, CD138- treatment groups.

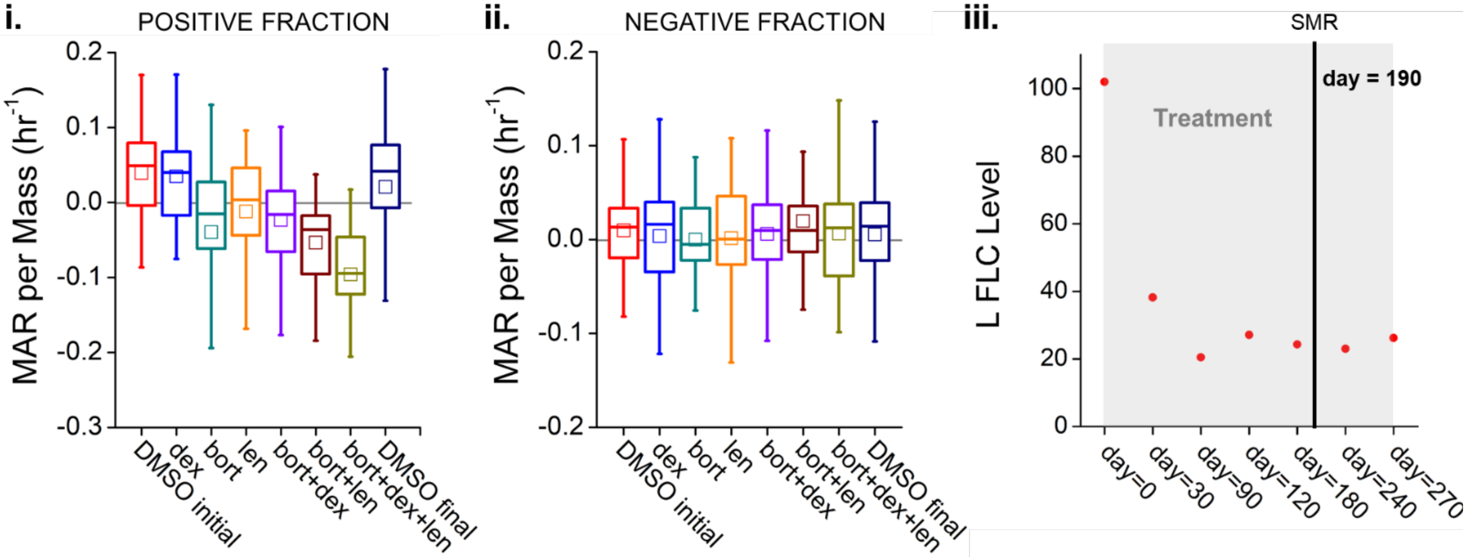

Supplementary Figure 14 Normalized MAR for control and treatment groups for (**i**) positive and (**ii**) negative fractions. Boxes represent the inter-quartile range and white squares are the average of all measurements. From left to right, number of cells in MAR measurement Positive: n = 64, 60, 64, 51, 50, 62, 54, 53 and Negative: n = 53, 65, 49, 58, 54, 49, 59, 61. (**iii**) L FLC level taken at different time points before and after the treatment.

PATIENT SAMPLE 7

Patient 7 (P7) was classified as "resistant" for dexamethasone, bortezomib and lenalidomide. The increase in the Kappa FLC level was observed within a 20 day-long period of treatment time with bortezomib + dexamethasone + lenalidomide. The MAR measurement was performed 28 days after the beginning of the treatment.

	BORT+DEX	BORT+LEN	FINAL
**p value**	1<br>(0.43)	1<br>(0.78)	1<br>(0.94)
**AUC**	0.51	0.51	0.53

Supplementary Table 9 Table of p and AUC values for Patient 7, CD138+ fraction treatment groups. Values calculated using initial control for comparison to each condition. p-values were determined by Welsh's t-test and Bonferroni corrected. Uncorrected p-value in parentheses.

	BORT+DEX	BORT+LEN	FINAL
**p value**	1<br>(0.37)	1<br>(0.69)	1<br>(0.59)
**AUC**	0.54	0.54	0.53

Supplementary Table 10 Table of p and AUC values for Patient 7, CD138- fraction treatment groups.

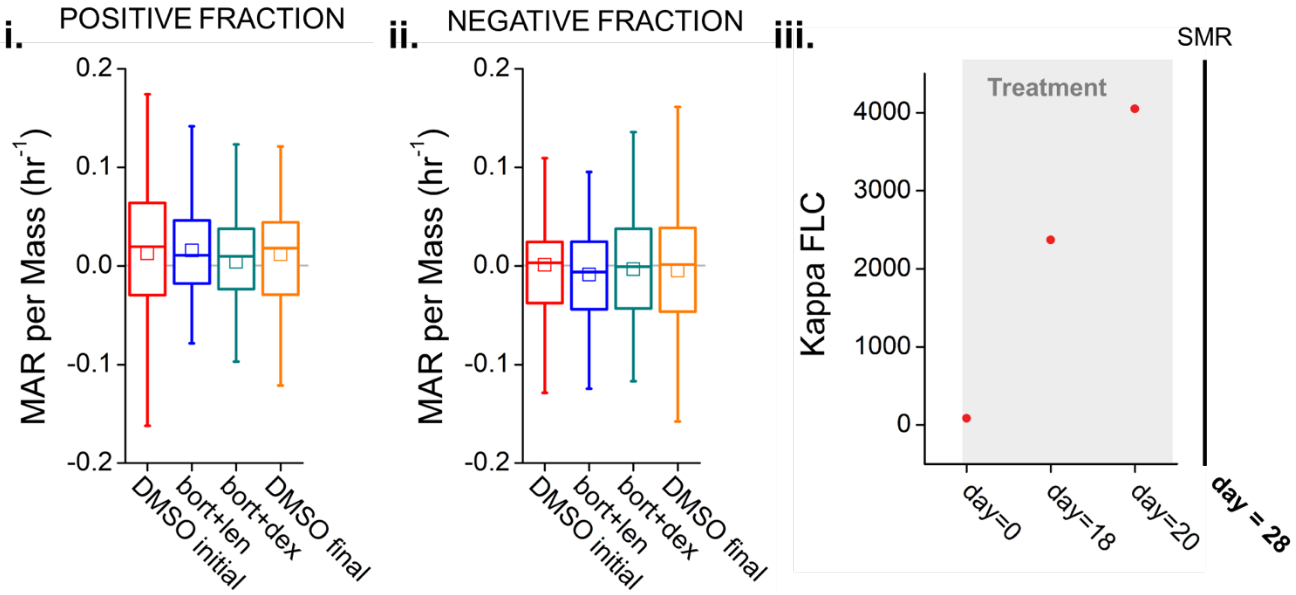

Supplementary Figure 15 Normalized MAR for control and treatment groups for (**i**) positive and (**ii**) negative fractions. Boxes represent the inter-quartile range and white squares are the average of all measurements. From left to right, number of cells in MAR measurement Positive: n = 69, 73, 71, 68 and Negative: n = 63, 67, 59, 75. (**iii**) Kappa FLC level taken at time points before and after the treatment.

PATIENT SAMPLE 8

Patient 8 (P8) was classified as "resistant" for dexamethasone, bortezomib and lenalidomide. The increase in the IgG level was observed within a 40 day-long period of treatment time with bortezomib + dexamethasone + lenalidomide. The MAR measurement was performed 15 days after the beginning of the treatment.

	DEX	BORT	LEN	BORT+DEX	BORT+LEN	FINAL
**p value**	1<br>(0.17)	0.092<br>(0.015)	1<br>(0.23)	0.35<br>(0.059)	0.088<br>(0.015)	1<br>(0.77)
**AUC**	0.58	0.65	0.59	0.63	0.66	0.53

Supplementary Table 11 Table of p and AUC values for Patient 8 treatment groups. Values initial control for comparison to each condition. p-values were determined by Welsh's t-test and Bonferroni corrected. Uncorrected p-value in parentheses.

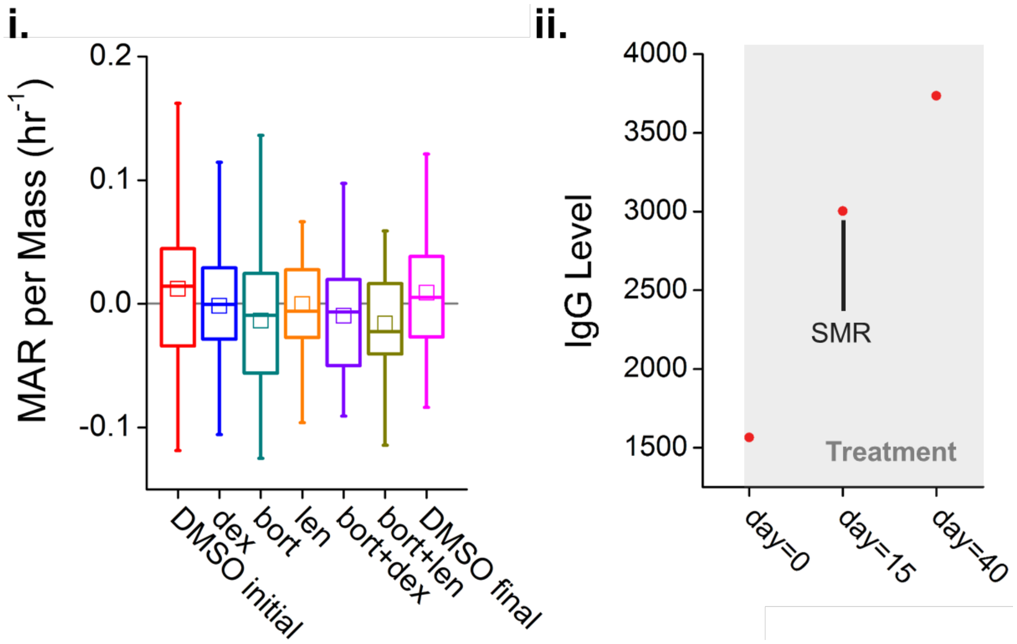

Supplementary Figure 16 (**i**) Normalized MAR for control and treatment groups. Boxes represent the inter-quartile range and white squares are the average of all measurements. From left to right, number of cells in MAR measurement n = 64, 60, 64, 51, 50, 62, 54. (**ii**) IgG level taken at different time points before and after the treatment.

PATIENT SAMPLE 9

Patient 79 (P9) was classified as "resistant" for dexamethasone, bortezomib and lenalidomide. The increase in the IgG level was observed 370 days after the treatment start date with bortezomib + dexamethasone + lenalidomide, when the MAR measurement was performed.

	DEX	BORT	LEN	BORT+DEX	BORT+LEN	BORT+DEX+LEN	FINAL
p value	1 (0.59)	1 (0.76)	1 (0.89)	0.96 (0.16)	1 (0.31)	1 (0.17)	0.9 (0.15)
AUC	0.55	0.56	0.52	0.58	0.58	0.58	0.58

Supplementary Table 12 Table of p and AUC values for Patient 9, CD138+ fraction treatment groups. Values calculated using using initial control for comparison to each condition. p-values were determined by Welsh's t-test and Bonferroni corrected. Uncorrected p-value in parentheses.

	DEX	BORT	LEN	BORT +DEX	BORT +LEN	BORT +DEX+LEN	FINAL
p value	1 (0.74)	1 (0.34)	1 (0.68)	1 (0.87)	1 (0.54)	1 (0.64)	1 (0.43)
AUC	0.52	0.53	0.52	0.51	0.52	0.54	0.52

Supplementary Table 13 Table of p and AUC values for Patient 9, CD138- fraction treatment groups.

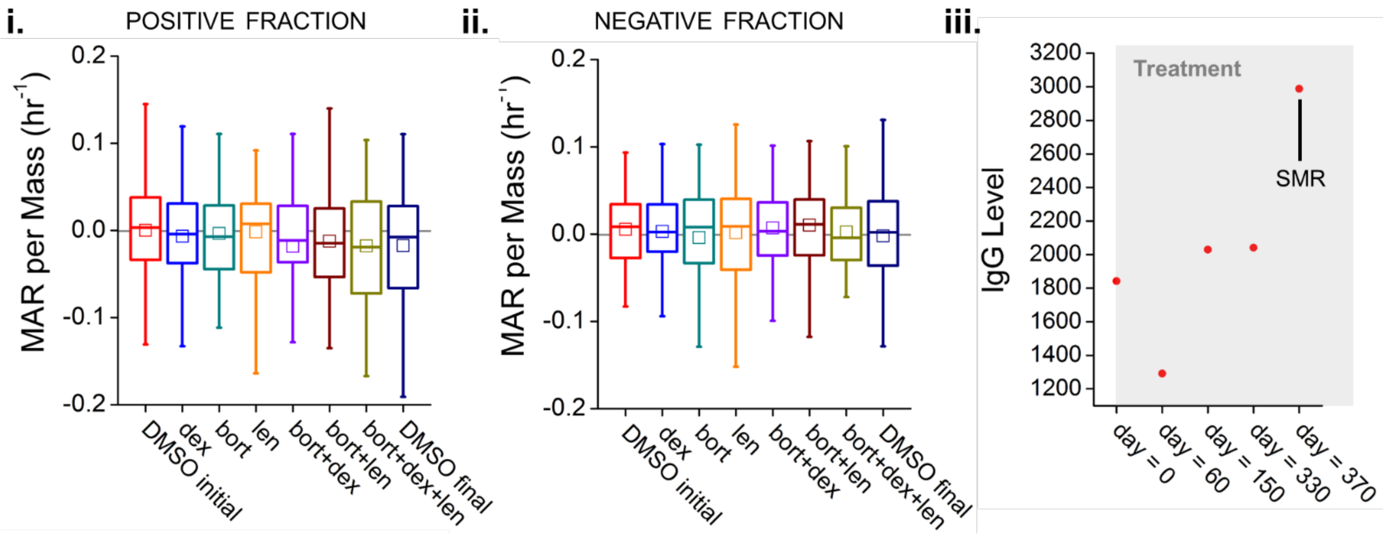

Supplementary Figure 17 Normalized MAR for control and treatment groups for (**i**) positive and (**ii**) negative fractions. Boxes represent the inter-quartile range and white squares are the average of all measurements. From left to right, number of cells in MAR measurement Positive: n = 51, 54, 54, 66, 54, 59, 54, 67 and Negative: n = 60, 59, 53, 54, 61, 62, 55, 65. (**iii**) IgG level taken at different time points before and after the treatment.

SUPPLEMENTARY REFERENCES

1. N. Cermak, S. Olcum, F. F. Delgado, S. C. Wasserman, KR Payer et al. High-throughput single-cell growth measurements via serial microfluidic mass sensor arrays, *Nat. Biotechnol.* **34,** 1052-1059 (2016).
2. J. Lee, R. Chunara, W. Shen, K. Payer, K. Babcock et al. Suspended microchannel resonators with piezoresistive sensors, *Lab. Chip.* **11,** 645-651 (2011).
3. T. P. Burg, M. Godin, S. M. Knudsen, W. Shen, G. Carlson et al. Weighing of biomolecules, single cells and single nanoparticles in fluid. *Nature* **446,** 1066-1069 (2007).
4. S. Olcum, N. Cermak, S. C. Wasserman, K. S. Christine, H. Atsumi et al. Weighing nanoparticles in solution at the attogram scale, *Proc. Natl. Acad. Sci. U.S.A.* **111,** 1310-1315 (2014).
5. S. Olcum, N. Cermak, S. C. Wasserman, S. R. Manalis, High-speed multiple-mode mass-sensing resolves dynamic nanoscale mass distributions. *Nat. Commun.* **6,** 7070 (2015).
