# Supplementary material for: Determining therapeutic susceptibility in multiple myeloma by single-cell mass accumulation
Source: Nat Commun. 2017 Nov 20;8:1613. doi: 10.1038/s41467-017-01593-2 (PMC5694762; doi:10.1038/s41467-017-01593-2)
Supplement: Supplementary file 2 — Peer Review File [file 41467_2017_1593_MOESM2_ESM.pdf]

Editorial Note: this manuscript has been previously reviewed at another journal that is not operating a transparent peer review scheme. This document only contains reviewer comments and rebuttal letters for versions considered at Nature Communications.

REVIEWERS' COMMENTS:

Reviewer #2 (Remarks to the Author):

The authors have responded appropriately to prior critiques, and significantly improved the manuscript. Clearly further studies in primary patient samples will be required to establish the positive and negative predictive value of this assay, which appears to be rapid and reproducible.

Reviewer #2:

Remarks to the Author:

1. The authors have responded appropriately to prior critiques, and significantly improved the manuscript. Clearly further studies in primary patient samples will be required to establish the positive and negative predictive value of this assay, which appears to be rapid and reproducible.

We appreciate the reviewers' comments, which we agree have considerably improved the manuscript. Our thanks to the reviewers for their high-quality input.
